# Supplementary material for: The Extracellular Bone Marrow Microenvironment—A Proteomic Comparison of Constitutive Protein Release by In Vitro Cultured Osteoblasts and Mesenchymal Stem Cells
Source: Cancers (Basel). 2020 Dec 28;13(1):62. doi: 10.3390/cancers13010062 (PMC7795818; doi:10.3390/cancers13010062)
Supplement: Supplementary file 1 [file cancers-13-00062-s001.pdf]

SUPPLEMENTARY INFORMATION

The extracellular bone marrow microenvironment – a proteomic comparison of constitutive protein release by *in vitro* cultured osteoblasts and mesenchymal stem cells

Elise Aasebø [1], Even Birkeland [2], Frode Selheim [2], Frode Berven [2], Annette K. Brenner [1] and Øystein Bruserud [1, 3,*]

[1] Department of Clinical Science, University of Bergen, Bergen, Norway; elise.aasebo@uib.no, annette.brenner@uib.no, oystein.bruserud@uib.no

[2] The Proteomics Facility of the University of Bergen (PROBE), University of Bergen, Bergen, Norway; even.birkeland@uib.no, frode.selheim@uib.no, frode.berven@uib.no

[3] Department of Medicine, Haukeland University Hospital, Bergen, Norway; oystein.bruserud@helse-bergen.no

Table S1. Proteins released by *in vitro* cultured MSCs and/or osteoblasts; a summary showing important groups of proteins. Only structural groups/main groups including at least five members are shown.

**EXTRACELLULAR MATRIX *(n=34)***	
**Collagens *(n=22)***	
COL1A1	Collagen alpha-1(I) chain
COL1A2	Collagen alpha-2(I) chain
COL2A1	Collagen alpha-1(II) chain;Collagen alpha-1(II) chain;Chondrocalcin
COL3A1	Collagen alpha-1(III) chain
COL4A1	Collagen alpha-1(IV) chain;Arresten
COL4A2	Collagen alpha-2(IV) chain;Canstatin
COL5A1	Collagen alpha-1(V) chain
COL5A2	Collagen alpha-2(V) chain
COL5A3	Collagen alpha-3(V) chain
COL6A1	Collagen alpha-1(VI) chain
COL6A2	Collagen alpha-2(VI) chain
COL6A2	Collagen alpha-2(VI) chain
COL6A3	Collagen alpha-3(VI) chain
COL7A1	Collagen alpha-1(VII) chain
COL8A1	Collagen alpha-1(VIII) chain;Vastatin
COL10A1	Collagen alpha-1(X) chain
COL11A1	Collagen alpha-1(XI) chain
COL12A1	Collagen alpha-1(XII) chain
COL14A1	Collagen alpha-1(XIV) chain
COL16A1	Collagen alpha-1(XVI) chain
COL18A1	Collagen alpha-1(XVIII) chain;Endostatin
COLGALT1	Procollagen galactosyltransferase 1
**Fibulin, fibrillin *(n=6)***	
FBLN1	Fibulin-1
FBLN1	Fibulin-1
FBLN2	Fibulin-2
FBLN5	Fibulin-5
FBN1	Fibrillin-1
FBN2	Fibrillin-2
**Laminin *(n=6)***	
LAMA1	Laminin subunit alpha-1
LAMA2	Laminin subunit alpha-2
LAMA4	Laminin subunit alpha-4
LAMB1	Laminin subunit beta-1
LAMB2	Laminin subunit beta-2
LAMC1	Laminin subunit gamma-1
**COMPLEMENT FACTORS *(n=11)***	
C1QBP	Complement component 1 Q
C1R	Complement C1r subcomponent
C1S	Complement C1s subcomponent
C2	Complement C2

C3	Complement C3
C4A	Complement C4-A
C4B	Complement C4-B
CFB	Complement factor B
CFD	Complement factor D
CFH	Complement factor H
CFI	Complement factor I

PROTEASES AND PROTEASE INHIBITORS *(n=26)*

Cathepsin proteases *(n=7)*

CTSD	Cathepsin D
CTSF	Cathepsin F
CTSG	Cathepsin G
CTSK	Cathepsin K
CTSL	Cathepsin L1
CTSS	Cathepsin S
CTSZ	Cathepsin Z

ADAM proteases (*n*=7)

ADAM9	Disintegrin and metalloproteinase domain-containing protein 9
ADAM10	Disintegrin and metalloproteinase domain-containing protein 10
ADAM19	Disintegrin and metalloproteinase domain-containing protein 19
ADAMTS1	A disintegrin and metalloproteinase with thrombospondin motifs 1
ADAMTS2	A disintegrin and metalloproteinase with thrombospondin motifs 2
ADAMTS4	A disintegrin and metalloproteinase with thrombospondin motifs 4
ADAMTSL1	ADAMTS-like protein 1

Serpins (protease inhibitors, *n=12*)

SERPINA9	Serpin A9
SERPINB1	Leukocyte elastase inhibitor
SERPINB10	Serpin B10
SERPINB2	Plasminogen activator inhibitor 2
SERPINB6	Serpin B6
SERPINB7	Serpin B7
SERPINB8	Serpin B8
SERPINE1	Plasminogen activator inhibitor 1
SERPINE2	Glia-derived nexin
SERPINF1	Pigment epithelium-derived factor
SERPING1	Plasma protease C1 inhibitor
SERPINH1	Serpin H1

CELL SURFACE MOLECULES *(n=13)*

Cadherins *(n=3)*

CDH11	Cadherin-11
CDH13	Cadherin-13
CDH2	Cadherin-2

HLA molecules *(n=4)*

HLA-A	HLA class I histocompatibility antigen

HLA-B	HLA class I histocompatibility antigen, alpha chain
HLA-C	HLA class I histocompatibility antigen, alpha chain
HLA-DRA	HLA class II histocompatibility antigen, DR alpha chain

Integrins *(n=5)*

ITGA5	Integrin alpha-5
ITGAM	Integrin alpha-M
ITGB1	Integrin beta-1
ITGB2	Integrin beta-2
ITGBL1	Integrin beta-like protein 1

INSULIN-LIKE GROWTH FACTOR *(n=9)*

IGF2	Insulin-like growth factor II
IGF2R	Cation-independent mannose-6-phosphate receptor
IGFALS	Insulin-like growth factor-binding protein complex acid labile subunit
IGFBP2	Insulin-like growth factor-binding protein 2
IGFBP3	Insulin-like growth factor-binding protein 3
IGFBP4	Insulin-like growth factor-binding protein 4
IGFBP5	Insulin-like growth factor-binding protein 5
IGFBP6	Insulin-like growth factor-binding protein 6
IGFBP7	Insulin-like growth factor-binding protein 7

GTPases, GTP BINDING PROTEINS, RAS-RELATED PROTEINS *(n=21)*

GTPases *(n=5)*

ARHGAP1	Rho GTPase-activating protein 1
ARHGAP30	Rho GTPase-activating protein 30
ARHGDIA	Rho GDP-dissociation inhibitor 1
ARHGDIB	Rho GDP-dissociation inhibitor 2
ARHGEF1	Rho guanine nucleotide exchange factor 1

Septins (GTP binding proteins, *n=5*)

SEPT2	Septin-2
SEPT6	Septin-6
SEPT7	Septin-7
SEPT9	Septin-9
SEPT11	Septin-11

RAS-related proteins (*n=11*)

RAB14	Ras-related protein Rab-14
RAB1A	Ras-related protein Rab-1A
RAB1B	Ras-related protein Rab-1B
RAB2A	Ras-related protein Rab-2A
RAB5C	Ras-related protein Rab-5C
RAB6A	Ras-related protein Rab-6A
RAB7A	Ras-related protein Rab-7A
RAB8B	Ras-related protein Rab-8B
RAB10	Ras-related protein Rab-10
RAB11A	Ras-related protein Rab-11A
RAB11B	Ras-related protein Rab-11B

PROTEIN SYNTHESIS, STABILIZATION AND MODULATION *(n=156)*

RNA binding proteins (*n=5*)

RBM3	RNA-binding protein 3
RBM8A	RNA-binding protein 8A
RBM25	RNA-binding protein 25
RBM42	RNA-binding protein 42
RBMX;RBMXL1	RNA-binding motif protein, X chromosome

Splicing factors *(n=12)*

SF3A1	Splicing factor 3A subunit 1
SF3A3	Splicing factor 3A subunit 3
SF3B1	Splicing factor 3B subunit 1
SF3B2	Splicing factor 3B subunit 2
SF3B3	Splicing factor 3B subunit 3
SF3B4	Splicing factor 3B subunit 4
SF3B5	Splicing factor 3B subunit 5
SFPQ	Splicing factor, proline- and glutamine-rich
SRSF1	Serine/arginine-rich splicing factor 1
SRSF2	Serine/arginine-rich splicing factor 2
SRSF6;SRSF4	Serine/arginine-rich splicing factor 6; Serine/arginine-rich splicing factor 4
SRSF7	Serine/arginine-rich splicing factor 7

Ribosomal proteins (*n=44*)

RPL3	60S ribosomal protein L3
RPL4	60S ribosomal protein L4
RPL5	60S ribosomal protein L5
RPL6	60S ribosomal protein L6
RPL7A	60S ribosomal protein L7a
RPL9	60S ribosomal protein L9
RPL10A	60S ribosomal protein L10a
RPL12	60S ribosomal protein L12
RPL13	60S ribosomal protein L13
RPL13A	60S ribosomal protein L13a
RPL15	60S ribosomal protein L15
RPL17	60S ribosomal protein L17
RPL18	60S ribosomal protein L18
RPL23	60S ribosomal protein L23
RPL23A	60S ribosomal protein L23a
RPL29	60S ribosomal protein L29
RPL30	60S ribosomal protein L30
RPLP0;RPLP0P6	60S acidic ribosomal protein P0
RPLP1	60S acidic ribosomal protein P1
RPLP2	60S acidic ribosomal protein P2
RPS2	40S ribosomal protein S2
RPS3	40S ribosomal protein S3
RPS11	40S ribosomal protein S11
RPS12	40S ribosomal protein S12

RPS13	40S ribosomal protein S13
RPS14	40S ribosomal protein S14
RPS15	40S ribosomal protein S15
RPS15A	40S ribosomal protein S15a
RPS17	40S ribosomal protein S17
RPS18	40S ribosomal protein S18
RPS19	40S ribosomal protein S19
RPS20	40S ribosomal protein S20
RPS21	40S ribosomal protein S21
RPS23	40S ribosomal protein S23
RPS26;RPS26P11	40S ribosomal protein S26
RPS28	40S ribosomal protein S28
RPS3A	40S ribosomal protein S3a
RPS4X;RPS4Y1	40S ribosomal protein S4, X isoform
RPS5	40S ribosomal protein S5
RPS6	40S ribosomal protein S6
RPS7	40S ribosomal protein S7
RPS8	40S ribosomal protein S8
RPSA	40S ribosomal protein SA
RRBP1	Ribosome-binding protein 1

Translation; initiation and elongation (*n=21*)

EIF1	Eukaryotic translation initiation factor 1
EIF2S1	Eukaryotic translation initiation factor 2 subunit 1
EIF2S2	Eukaryotic translation initiation factor 2 subunit 2
EIF2S3;EIF2S3L	Eukaryotic translation initiation factor 2 subunit 3
EIF3C;EIF3CL	Eukaryotic translation initiation factor 3 subunit C
EIF3G	Eukaryotic translation initiation factor 3 subunit G
EIF3H	Eukaryotic translation initiation factor 3 subunit H
EIF3I	Eukaryotic translation initiation factor 3 subunit I
EIF3J	Eukaryotic translation initiation factor 3 subunit J
EIF3M	Eukaryotic translation initiation factor 3 subunit M
EIF4A1;EIF4A2	Eukaryotic initiation factor 4A-I
EIF4A3	Eukaryotic initiation factor 4A-III
EIF4B	Eukaryotic translation initiation factor 4B
EIF4G1	Eukaryotic translation initiation factor 4 gamma 1
EIF5A;EIF5AL1	Eukaryotic translation initiation factor 5A-1
EIF6	Eukaryotic translation initiation factor 6
EEF1A1;EEF1A1P5;EEF1A2	Elongation factor 1-alpha 1
EEF1B2	Elongation factor 1-beta
EEF1E1	Eukaryotic translation elongation factor 1 epsilon-1
EEF1G	Elongation factor 1-gamma
EEF2	Elongation factor 2

Chaperones: T complex proteins (*n=7*)

CCT2	T-complex protein 1 subunit beta
CCT3	T-complex protein 1 subunit gamma
CCT4	T-complex protein 1 subunit delta
CCT5	T-complex protein 1 subunit epsilon

CCT6A	T-complex protein 1 subunit zeta
CCT7	T-complex protein 1 subunit eta
CCT8	T-complex protein 1 subunit theta
**Heat shock protein (*n=17*)**	
HSBP1	Heat shock factor-binding protein 1
HSP90AA1	Heat shock protein HSP 90-alpha
HSP90AA4P	Putative heat shock protein HSP 90-alpha A4
HSP90AB1	Heat shock protein HSP 90-beta
HSP90AB2P	Putative heat shock protein HSP 90-beta 2
HSP90AB4P	Putative heat shock protein HSP 90-beta 4
HSP90B1	Endoplasmin
HSPA1B;HSPA1A	Heat shock 70 kDa protein 1B;
HSPA4	Heat shock 70 kDa protein 4
HSPA5	78 kDa glucose-regulated protein
HSPA6;HSPA7	Heat shock 70 kDa protein 6
HSPA8	Heat shock cognate 71 kDa protein
HSPA9	Stress-70 protein, mitochondrial
HSPB1	Heat shock protein beta-1
HSPD1	60 kDa heat shock protein, mitochondrial
HSPE1	10 kDa heat shock protein, mitochondrial
HSPH1	Heat shock protein 105 kDa
**Peptidyl-prolyl cis-trans (*n=6*)**	
FKBP1A	Peptidyl-prolyl cis-trans isomerase FKBP1A
FKBP3	Peptidyl-prolyl cis-trans isomerase FKBP3
FKBP4	Peptidyl-prolyl cis-trans isomerase FKBP4
FKBP5	Peptidyl-prolyl cis-trans isomerase FKBP5
FKBP9	Peptidyl-prolyl cis-trans isomerase FKBP9
FKBP10	Peptidyl-prolyl cis-trans isomerase FKBP10
**Glycosylation (*n=4*)**	
GALNT2	Polypeptide N-acetylgalactosaminyltransferase 2
GALNT5	Polypeptide N-acetylgalactosaminyltransferase 5
GALNT6	Polypeptide N-acetylgalactosaminyltransferase 6
GALNT16	Polypeptide N-acetylgalactosaminyltransferase 16
**Ubiquitination (*n=12*)**	
UBE2K	Ubiquitin-conjugating enzyme E2 K
UBE2L3	Ubiquitin-conjugating enzyme E2 L3
UBE2N	Ubiquitin-conjugating enzyme E2 N
UBE2V1	Ubiquitin-conjugating enzyme E2 variant 1
UBQLN1;UBQLN2	Ubiquilin-1;Ubiquilin-2
UCHL1	Ubiquitin carboxyl-terminal hydrolase isozyme L1
UCHL3	Ubiquitin carboxyl-terminal hydrolase isozyme L3
UFM1	Ubiquitin-fold modifier 1
USP14	Ubiquitin carboxyl-terminal hydrolase 14
USP15	Ubiquitin carboxyl-terminal hydrolase 15
USP48	Ubiquitin carboxyl-terminal hydrolase 48
USP5	Ubiquitin carboxyl-terminal hydrolase 5

Proteasomal proteins ***(n=20)***

PSMA1	Proteasome subunit alpha type-1
PSMA2	Proteasome subunit alpha type-2
PSMA3	Proteasome subunit alpha type-3
PSMA4	Proteasome subunit alpha type-4
PSMA5	Proteasome subunit alpha type-5
PSMA6	Proteasome subunit alpha type-6
PSMA7	Proteasome subunit alpha type-7
PSMB1	Proteasome subunit beta type-1
PSMB2	Proteasome subunit beta type-2
PSMB3	Proteasome subunit beta type-3
PSMB4	Proteasome subunit beta type-4
PSMB5	Proteasome subunit beta type-5
PSMB6	Proteasome subunit beta type-6
PSMB7	Proteasome subunit beta type-7
PSMB8	Proteasome subunit beta type-8
PSMB9	Proteasome subunit beta type-9
PSMB10	Proteasome subunit beta type-10
PSME1	Proteasome activator complex subunit 1
PSME2	Proteasome activator complex subunit 2
PSME3	Proteasome activator complex subunit 3

Protein phosphorylation ***(n=8)***

PPP1CA;PPP1CC	Serine/threonine-protein phosphatase PP1-alpha catalytic subunit;
PPP1R14B	Protein phosphatase 1 regulatory subunit 14B
PPP1R18	Phostensin
PPP1R7	Protein phosphatase 1 regulatory subunit 7
PPP2CA; PPP2CB	Serine/threonine-protein phosphatase 2A catalytic subunit alpha isoform
PPP2R1A	Serine/threonine-protein phosphatase 2A 65 kDa regulatory subunit A alpha isoform
PPP3CA	Serine/threonine-protein phosphatase 2B catalytic subunit alpha isoform
PPP3R1	Calcineurin subunit B type 1

CYTOSKELETON ***(n=22)***

Actin and actin-interacting proteins ***(n=19)***

ACTB	Actin, cytoplasmic 1
ACTBL2	Beta-actin-like protein 2
ACTC1	Actin, alpha cardiac muscle 1
ACTG1	Actin, cytoplasmic 2
ACTL6A	Actin-like protein 6A
ACTN1	Alpha-actinin-1
ACTN4	Alpha-actinin-4
ACTR1A	Alpha-centractin
ACTR2	Actin-related protein 2
ACTR3	Actin-related protein 3
CAPZA1	F-actin-capping protein subunit alpha-1
CAPZA2	F-actin-capping protein subunit alpha-2

CAPZB	F-actin-capping protein subunit beta
ARPC1A	Actin-related protein 2/3 complex subunit 1A
ARPC1B	Actin-related protein 2/3 complex subunit 1B
ARPC2	Actin-related protein 2/3 complex subunit 2
ARPC3	Actin-related protein 2/3 complex subunit 3
ARPC4	Actin-related protein 2/3 complex subunit 4
ARPC5	Actin-related protein 2/3 complex subunit 5

Tropomyosin *(n=3)*

TPM1	Tropomyosin alpha-1 chain
TPM3	Tropomyosin alpha-3 chain
TPM4	Tropomyosin alpha-4 chain

ORGANELLAR FUNCTIONS - V-ATPases (*n*=9)

ATP5A1	ATP synthase subunit alpha, mitochondrial
ATP5B	ATP synthase subunit beta, mitochondrial
ATP5D	ATP synthase subunit delta, mitochondrial
ATP5J	ATP synthase-coupling factor 6, mitochondrial
ATP6AP1	V-type proton ATPase subunit S1
ATP6AP2	Renin receptor
ATP6V1A	V-type proton ATPase catalytic subunit A
ATP6V1B2	V-type proton ATPase subunit B
ATP6V1E1	V-type proton ATPase subunit E 1

NUCLEAR PROTEINS *(n=49)*

Histones *(n=18)*

H1F0	Histone H1.0;Histone H1.0, N-terminally processed
H2AFV;H2AFZ	Histone H2A.V;Histone H2A.Z
H2AFY	Core histone macro-H2A.1
H3F3A	Histone H3.3
HIST1H1B	Histone H1.5
HIST1H1C	Histone H1.2
HIST1H1D	Histone H1.3
HIST1H1E	Histone H1.4
HIST1H2AC	Histone H2A type 1-C;Histone H2A type 3
HIST1H2AJ	Histone H2A type 1-J
HIST1H2BM	Histone H2B type 1-M
HIST1H3A	Histone H3.1;Histone H3.1t;Histone H3.3C
HIST1H4A	Histone H4
HIST2H2AC	Histone H2A type 2-C;Histone H2A type 2-A
HIST2H2BE	Histone H2B type 2-E;Histone H2B type 1-B
HIST2H3A	Histone H3.2
RBBP4	Histone-binding protein RBBP4
RBBP7	Histone-binding protein RBBP7

Non-histone chromatin proteins *(n=20)*

HMGN1	Non-histone chromosomal protein HMG-14
HMGN2	Non-histone chromosomal protein HMG-17
HNRNPA0	Heterogeneous nuclear ribonucleoprotein A0

HNRNPA1;HNRNPA1L2	Heterogeneous nuclear ribonucleoprotein A1
HNRNPA2B1	Heterogeneous nuclear ribonucleoproteins A2/B1
HNRNPA3	Heterogeneous nuclear ribonucleoprotein A3
HNRNPAB	Heterogeneous nuclear ribonucleoprotein A/B
HNRNPC	Heterogeneous nuclear ribonucleoproteins C1/C2
HNRNPD	Heterogeneous nuclear ribonucleoprotein D0
HNRNPDL	Heterogeneous nuclear ribonucleoprotein D-like
HNRNPF	Heterogeneous nuclear ribonucleoprotein F
HNRNPH1	Heterogeneous nuclear ribonucleoprotein H
HNRNPH3	Heterogeneous nuclear ribonucleoprotein H3
HNRNPK	Heterogeneous nuclear ribonucleoprotein K
HNRNPL	Heterogeneous nuclear ribonucleoprotein L
HNRNPM	Heterogeneous nuclear ribonucleoprotein M
HNRNPR	Heterogeneous nuclear ribonucleoprotein R
HNRNPU	Heterogeneous nuclear ribonucleoprotein U
HNRNPUL2	Heterogeneous nuclear ribonucleoprotein U-like protein 2
HP1BP3	Heterochromatin protein 1-binding protein 3
**Nuclear proteins *(n=11)***	
SNRNP200	U5 small nuclear ribonucleoprotein 200 kDa helicase
SNRPA	U1 small nuclear ribonucleoprotein A
SNRPA1	U2 small nuclear ribonucleoprotein A
SNRPB2	U2 small nuclear ribonucleoprotein B
SNRPD1	Small nuclear ribonucleoprotein Sm D1
SNRPD2	Small nuclear ribonucleoprotein Sm D2
SNRPD3	Small nuclear ribonucleoprotein Sm D3
SNRPE	Small nuclear ribonucleoprotein E
SNRPF	Small nuclear ribonucleoprotein F
SNRPG	Small nuclear ribonucleoprotein G
SRRM1	Serine/arginine repetitive matrix protein 1
**INTRACELLULAR TRANSPORT *(n=14)***	
**14-3-3 proteins *(n=6)***	
YWHAB	14-3-3 protein beta/alpha;14-3-3 protein beta/alpha
YWHAE	14-3-3 protein epsilon
YWHAG	14-3-3 protein gamma;14-3-3 protein gamma
YWHAH	14-3-3 protein eta
YWHAQ	14-3-3 protein theta
YWHAZ	14-3-3 protein zeta/delta
**SLC transporter *(n=8)***	
SLC1A5	Neutral amino acid transporter B(0)
SLC25A5	ADP/ATP translocase 2;ADP/ATP translocase 2, N-terminally processed
SLC25A6;SLC25A4	ADP/ATP translocase 3
SLC2A1	Solute carrier family 2, facilitated glucose transporter member 1
SLC38A2	Sodium-coupled neutral amino acid transporter 2
SLC3A2	4F2 cell-surface antigen heavy chain
SLC4A1	Band 3 anion transport protein

SLC9A3R1	Na(+)/H(+) exchange regulatory cofactor NHE-RF1

METABOLISM, REDOX BALANCE *(n=10)*

Lipoproteins *(n=4)*

APOB	Apolipoprotein B-100
APOBR	Apolipoprotein B receptor
APOC3	Apolipoprotein C-III
APOE	Apolipoprotein E

Peroxiredoxins (antioxidant, *n=6)*

PRDX1	Peroxiredoxin-1
PRDX2	Peroxiredoxin-2
PRDX3	Thioredoxin-dependent peroxide reductase, mitochondrial
PRDX4	Peroxiredoxin-4
PRDX5	Peroxiredoxin-5, mitochondrial
PRDX6	Peroxiredoxin-6

S100 PROTEINS *(n=8)*

S100A4	Protein S100-A4
S100A6	Protein S100-A6
S100A9	Protein S100-A9
S100A10	Protein S100-A10
S100A11	Protein S100-A11
S100A12	Protein S100-A12;Calcitermin
S100A13	Protein S100-A13
S100P	Protein S100-P

Table S2. Extracellular protein release by osteoblasts and bone marrow MSCs. The table presents a classification of 163 detected proteins belonging to the GO term Extracellular matrix. The proteins were either released by both cell types, showed increased levels in MSCs (marked with blue) or increased levels in osteoblasts (marked with green).

**EXTRACELLULAR MATRIX AND MATRICELLULAR MOLECULES *(n=72)***
**Collagen (*n*=20):** COL1A1, COL1A2, COL2A1, COL3A1, COL4A1, COL4A2, COL5A1, COL5A2, COL5A3, COL6A1, COL6A2, COL6A3, COL7A1, COL8A1, COL10A1, COL11A1, COL12A1, COL14A1, COL16A1, COL18A1.
**Fibulin *(n=5)*:** N1, FBLN2, FBLN5, EFEMP1, EFEMP2.
**Fibrillin *(n=2)*:** FBN1, FBN2.
**Laminin *(n=6)*:** LAMA1, LAMA2, LAMA4, LAMB2, LAMB1, LAMC1.
**Galectin *(n=2)*:** LGASL1, LGALS3, LGALS3BP.
**Thrombospondin *(n=4)*:** THBS1, THBS2, THBS3, THBS4.
**Other matrix molecules (*n*=33):** ABI3BP, ACAN, AGR, BGN, CSPG4, DCN, ECM1, ECM2, ELN, EMILIN1, FCN1, FMOD, FN1, HAPLN1, HMCN1, HSPG2, LOXL3, LUM, MFAP2, MFAP5, MFGE8, MGP, NID1, NID2, OGN, OMD, MXRA5, NDNF, PODN, POSTN, PRELP, PRG4, SPARC3, TNC,VCAN.
**PROTEASES, PROTEASE ENHANCERS *(n=14)***
ADAM9, ADAM10, ADAM19, ADAMTS1, ADAMTS2, ADAMTS4, ELANE, MMP2, MMP1, MMP8, MMP9, MMP11, MMP14, HTRA1, PCOLCE, PRTN3.
**PROTEASE INHIBITORS *(n=16)***
**Serpins *(n=8)*:** SERPINB1, SERPINB6, SERPINB8, SERPIN1, SERPIN2, SERPINF1, SERPING1, SERPINH1.
**Cathepsins *(n=6)*:** CTSB, CTSC, CTSD, CTSG, CTSS, CTSZ.
**Other protease inhibitors (*n*=2):** TIMP1, TIMP2.
**OTHER ENZYMES *(n=7)***
LOX, LOX1, LOX2, PKM, PLOD3, SOD3, SULF1
**CYTOKINES, SOLUBLE MEDIATORS *(n=13)***
**Cytokines** *(n=6)***:** ANGPTL2, ANXA2, CLC, HPX, HDGF, S100A8
**Cytokine modulators** (*n*=7)**:** IGFALS, IGFBP7, LPBP1, LTBP2, LTBP3, TGFB1, TNFRSF11B
**CELL SURFACE MEMBRANE *(n=16)***
**Adhesion molecules** *(n=4)***:** CD248, FLRT2, GPC1, NCAM1.
**Other cell surface membrane molecules or secreted mediators** *(n=12)***:** ALPL, ANXA1, ANXA5, CDH13, CDH14, COLEC12, GP1BA, LRRC15, PXDN, SEMA7A, SSC5D, VASN.
**SECRETION, EXOCYTOSIS, EXOSOMES *(n=3)***
ANXA6, CLEC3B, S100A6
**OTHER MOLECULES *(n=21)***
AEBP1 (transcription), APOE (metabolism), CALR (endoplasmatic reticulum), CCDC80 (peroxiredoxin?), CHI3L1, CLU (chaperone), CTH3C1, EDIL3, F13A1 (coagulation), HSP90B1 (chaperone), GREM1 (BMP antagonist), HNRNPM (nuclear RNA binding), PSAP (lysosome), PTX3, S100A10 (cytoplasm-nucleus), S100A9 (casein kinase modulator), SMOC1, SPON2, TGFB1 (enzyme), TGM2 (enzyme), VASP, WNT5B (Wnt signaling).

Table S3. Constitutive protein release by *in vitro* cultured MSCs derived from 10 donors; an overview of 426 proteins reaching detectable levels for at least nine of the 10 MSC donors.

ABI3BP	CDH11	EEF2	HNRNPA1	MCAM	PLXDC2	RSU1	TUBB2A/B
ACLY	CDH13	EFEMP2	HNRNPA2B1	MDH1	PLXNB2	RTN4	TUBB4A/B
ACTB	CDH2	EIF5A	HNRNPA3	MDH2	PNP	S100A11	TWF2
ACTC1	CFL1	EIF6	HNRNPK	MFGE8	POSTN	SAMHD1	TXN
ACTG1	CLEC11A	EMILIN1	HSP90AB1	MMP1	POSTN	SDC1	TXNDC5
ACTN1	CLIC1	ENO1	HSP90B1	MMP14	PPIA	SDF4	TXNL1
ACTN4	CLIC4	ENPP2	HSP90AA1	MMP2	PPIB	SEMA7A	TXNRD1
ACTR2	CLSTN1	ERH	HSPA1B	MRC2	PPP1R14B	SEPT11	UBE2L3
ACTR3	CLTC	ERP29	HSPA4	MSN	PPP2R1A	SERBP1	UBE2V1
ADAM10	CLU	EXT1	HSPA5	MTPN	PRDX1	SERPINB6	UCHL1
ADAM9	CNDP2	EXTL2	HSPA8	MVP	PRDX2	SERPINE1	UGP2
ADAMTSL1	CNN3	EZR	HSPA9	MXRA8	PRDX3	SERPINE2	ULBP2
ADH5	COL10A1	FABP5	HSPD1	MYH9	PRDX6	SERPINF1	VASN
AEBP1	COL11A1	FAM129B	HSPE1	MYL12A/B	PRSS23	SERPINH1	VCAN
AHNAK	COL12A1	FAP	HSPG2	MYL6	PSAP	SET	VCL
AK2	COL16A1	FASN	HTRA1	NAGK	PSAT1	SF3B1	VCP
AKAP12	COL18A1	FBLN1	IGFBP3	NAMPT	PSMA1	SH3BGRL	VIM
AKR1A1	COL1A1	FBLN1	IGFBP4	NCL	PSMA2	SH3BGRL3	WDR1
AKR1B1	COL1A2	FBLN5	IGFBP6	NEDD8	PSMA3	SLC3A2	XYLT1
ALCAM	COL3A1	FBN1	IGFBP7	NID1	PSMA4	SMOC1	YBX1
ALDOA	COL4A1	FH	ILF2	NID2	PSMA5	SOD1	YWHAB
ANP32A	COL4A2	FKBP1A	ILF3	NME1	PSMA6	SPARC	YWHAE
ANPEP	COL5A1	FLNA	INHBA	NME2	PSMA7	SPOCK1	YWHAG
ANTXR1	COL5A2	FLNB	IGF2	NPC2	PSMB1	SPON2	YWHAQ
ANXA2	COL6A1	FN1	IQGAP1	NPM1	PSMB2	SPTAN1	YWHAZ
ANXA6	COL6A2	FN1	IRF2BP2	NQO1	PSMB3	SRGN	ZYX
APEX1	COL6A3	FSCN1	ISLR	NRP1	PSMB5	SRPX	
APLP2	COL7A1	FSTL1	ITGA5	NRP2	PSMB6	SRPX2	
APP	COL8A1	GALNT10	ITGBL1	NT5E	PSMB7	STC1	
ARHGDIA	COLEC12	GALNT2	ITM2B	NUCB1	PTBP1	STC2	
ARPC1B	COMP	GALNT5	KPNB1	NUCB2	PTGDS	STMN1	
ARPC2	COTL1	GANAB	KRT6B	NUTF2	PTK7	SYNCRIP	
ARPC4	CPA4	GAPDH	LAMA4	OLFML2B	PTMA	TAGLN	
ATP5B	CPPED1	GAS6	LAMB1	OLFML3	PTPRG	TAGLN2	
ATP6AP1	CSF1	GDI2	LAMC1	P4HB	PTPRK	TF	
ATP6AP2	CSPG4	GGH	LCP1	PABPN1	PTX3	TGFBI	
B2M	CSRP1	GOLM1	LDHA	PAM	PXDN	TGOLN2	
BASP1	CST3	GPC1	LDHB	PARK7	QSOX1	THBS1	
BGN	CTHRC1	GPI	LGALS1	PCMT1	RAD23B	THBS2	
BLMH	CTSB	GREM1	LGALS3BP	PCOLCE	RAN	TIMP1	
BMP1	CTSD	GRN	LMAN2	PDIA3	RCN1	TIMP2	
C1QBP	CTSL	GSN	LMNA	PDIA4	RCN3	TKT	
C1R	CTSZ	GSTO1	LMNB1	PDLIM5	RNASET2	TLN1	
C1S	CYR61	GSTP1	LOX	PEBP1	RNH1	TMOD3	
CALD1	DAG1	HAPLN1	LOXL2	PFN1	RPL12	TMSB10	
CALR	DBN1	HARS	LRP1	PGAM1	RPL17	TMSB4X	
CALU	DCN	HCRTR1	LRRFIP1	PGD	RPL30	TP53I3	
CANT1	DDB1	HDGF	LSM8	PGK1	RPL4	TPI1	
CAP1	DKK3	HEXB	LTA4H	PGLS	RPLP0	TPM3	
CAPG	DNAH6	HIST1H1C	LTBP1	PGM2	RPLP1	TPM4	
CAPZA1	DPP3	HIST1H2AJ	LTBP2	PKM	RPLP2	TPP1	
CAT	DPYSL2	HIST1H4A	LUM	PLAU	RPS12	TPT1	
CBX3	ECM1	HIST2H2AC	MAN1A1	PLEC	RPS27A	TRIM28	
CCDC80	EEF1A1/2	HLA-A	MAN1B1	PLOD1	RPS28	TSN	
CD109	EEF1B2	HLA-B	MAP1B	PLOD2	RPS3	TSSK4	
CD248	EEF1D	HMGA1	MAP4	PLOD3	RPSA	TTN	
CDC37	EEF1G	HMGN2	MARCKS	PLTP	RRBP1	TUBA1A/B	

Table S4. Constitutive protein release by *in vitro* cultured osteoblasts derived from 9 donors; an overview of 317 proteins reaching detectable levels for at least eight of the nine osteoblast donors.

ABI3BP	CLEC11A	FKBP1A	HSPA8	NME1	PSMB1	TAGLN	ZYX
ACAN	CLEC3B	FLNA	HSPB1	NME2	PSMB2	TAGLN2	
ACTB	CLSTN1	FLNB	HSPD1	NPC2	PSMB6	TALDO1	
ACTC1	CLU	FMOD	HSPG2	NPM1	PSME1	TBCA	
ACTG1	COL11A1	FN1	HTRA1	NRP1	PTK7	TF	
ACTN1	COL12A1	FSCN1	IGF2R	NUCB1	PTMA	TGFBI	
ACTN4	COL1A1	FST	IGFBP3	OLFML3	PTMS	THBS1	
ACTR3	COL1A2	FSTL1	IGFBP4	P4HB	PTX3	THBS2	
ADAM10	COL3A1	GALNT2	IGFBP6	PAM	PXDN	TIMP1	
ADAM9	COL4A2	GANAB	IGFBP7	PARK7	QSOX1	TIMP2	
AEBP1	COL5A1	GAPDH	INHBA	PCOLCE	RAN	TKT	
AHCY	COL5A2	GAS6	IQGAP1	PDIA3	RCN1	TLN1	
AHNAK	COL6A1	GDI2	ISLR	PDIA4	RCN3	TMSB10	
AKR1A1	COL6A2	GOLM1	LAMA4	PDIA6	RNASET2	TMSB4X	
ALDOA	COL6A2	GPC1	LAMB1	PEBP1	RNH1	TPI1	
ANP32A	COL6A3	GPI	LAMC1	PFN1	RPL10A	TPM1	
ANPEP	COMP	GREM1	LASP1	PGAM1	RPL12	TPM3	
ANXA2	CORO1C	GSN	LCP1	PGD	RPLP0	TPM4	
APOB	COTL1	GSTO1	LDHA	PGK1	RPLP1	TPP1	
APOC3	CPPED1	GSTP1	LDHB	PGLS	RPLP2	TPT1	
APP	CSRP1	GAA	LGALS1	PKM	RPS27A	TSSK4	
ARHGDIA	CST3	HBA1	LGALS3BP	PLEC	RPS3	TTN	
ARPC1B	CTGF	HBB	LMNA	PLOD1	RTN4	TUBA1B	
ARPC2	CTSA	HDGF	LOX	PLOD2	S100A11	TUBB	
ARPC4	CTSB	HEXA	LOXL2	PLOD3	SDF4	TXN	
ATP5B	CTSD	HEXB	LRP1	PNP	SEMA7A	TXNDC5	
B2M	CTSL	HIST1H3A	LTA4H	POSTN	SERBP1	TXNRD1	
BASP1	CTSZ	HIST1H4A	LTBP1	PPIA	SERPINB1	UBA1	
BGN	DAG1	HIST2H2AC	LTBP2	PPIB	SERPINE1	UBE2V1	
C1R	DAP	HLA-A	LTF	PRCP	SERPINE2	UCHL1	
C1S	DCN	HLA-C	LUM	PRDX1	SERPINF1	VASN	
CALD1	DPP3	HMGA1	MAP4	PRDX2	SERPING1	VCAM1	
CALR	DPYSL2	HMGN2	MARCKS	PRDX3	SERPINH1	VCAN	
CALU	ECM1	HNRNPA1	MDH1	PRDX6	SET	VCL	
CAP1	EEF1A1	HNRNPA2B1	MDH2	PRKCSH	SH3BGRL3	VCP	
CAPG	EEF2	HNRNPA3	MFGE8	PROCR	SNRPD2	VIM	
CAT	EFEMP1	HNRNPD	MMP2	PROS1	SPARC	WDR1	
CCDC80	EFEMP2	HNRNPK	MSN	PSAP	SPOCK1	XYLT1	
CD109	EIF5A	HSP90AB1	MT2A	PSMA1	SPTAN1	YBX1	
CD248	EMILIN1	HSP90B1	MYH9	PSMA2	SPTBN1	YWHAB	
CD44	ENO1	HSP90AA1	MYL6	PSMA3	SRGN	YWHAE	
CDH11	ENPP2	HSPA1B	MYO18B	PSMA4	STC2	YWHAG	
CDH13	FBLN1	HSPA4	NCL	PSMA5	STMN1	YWHAH	
CFD	FBLN1	HSPA5	NID1	PSMA6	SUMO2	YWHAQ	
CFL1	FBN1	HSPA6	NID2	PSMA7	SYNCRIP	YWHAZ	

Table S5. Constitutive protein release by *in vitro* cultured MSCs derived from 10 donors and osteoblasts derived from 9 donors; an overview of 269 common proteins reaching detectable levels for at least nine MSC and eight osteoblast donors.

ABI3BP	COL1A1	GOLM1	LOX	POSTN	SH3BGRL3	YWHAG
ACTB	COL1A2	GPC1	LOXL2	PPIA	SPARC	YWHAQ
ACTC1	COL3A1	GPI	LRP1	PPIB	SPOCK1	YWHAZ
ACTG1	COL4A2	GREM1	LTA4H	PRDX1	SPTAN1	ZYX
ACTN1	COL5A1	GSN	LTBP1	PRDX2	SRGN	
ACTN4	COL5A2	GSTO1	LTBP2	PRDX3	STC2	
ACTR3	COL6A1	GSTP1	LUM	PRDX6	STMN1	
ADAM10	COL6A2	HDGF	MAP4	PSAP	SYNCRIP	
ADAM9	COL6A3	HEXB	MARCKS	PSMA1	TAGLN	
AEBP1	COMP	HIST1H4A	MDH1	PSMA2	TAGLN2	
AHNAK	COTL1	HIST2H2AC	MDH2	PSMA3	TF	
AKR1A1	CPPED1	HLA-A	MFGE8	PSMA4	TGFBI	
ALDOA	CSRP1	HMGA1	MMP2	PSMA5	THBS1	
ANP32A	CST3	HMGN2	MSN	PSMA6	THBS2	
ANPEP	CTSB	HNRNPA1	MYH9	PSMA7	TIMP1	
ANXA2	CTSD	HNRNPA2B1	MYL6	PSMB1	TIMP2	
APP	CTSL	HNRNPA3	NCL	PSMB2	TKT	
ARHGDIA	CTSZ	HNRNPK	NID1	PSMB6	TLN1	
ARPC1B	DAG1	HSP90AB1	NID2	PTK7	TMSB10	
ARPC2	DCN	HSP90B1	NME1	PTMA	TMSB4X	
ARPC4	DPP3	HSP90AA1	NME2	PTX3	TPI1	
ATP5B	DPYSL2	HSPA1B	NPC2	PXDN	TPM3	
B2M	ECM1	HSPA4	NPM1	QSOX1	TPM4	
BASP1	EEF1A1	HSPA5	NRP1	RAN	TPP1	
BGN	EEF2	HSPA8	NUCB1	RCN1	TPT1	
C1R	EFEMP2	HSPD1	OLFML3	RCN3	TSSK4	
C1S	EIF5A	HSPG2	P4HB	RNASET2	TTN	
CALD1	EMILIN1	HTRA1	PAM	RNH1	TUBA1B	
CALR	ENO1	IGFBP3	PARK7	RPL12	TUBB	
CALU	ENPP2	IGFBP4	PCOLCE	RPLP0	TXN	
CAP1	FBLN1	IGFBP6	PDIA3	RPLP1	TXNDC5	
CAPG	FBLN1	IGFBP7	PDIA4	RPLP2	TXNRD1	
CAT	FBN1	INHBA	PEBP1	RPS27A	UBE2V1	
CCDC80	FKBP1A	IQGAP1	PFN1	RPS3	UCHL1	
CD109	FLNA	ISLR	PGAM1	RTN4	VASN	
CD248	FLNB	LAMA4	PGD	S100A11	VCAN	
CDH11	FN1	LAMB1	PGK1	SDF4	VCL	
CDH13	FSCN1	LAMC1	PGLS	SEMA7A	VCP	
CFL1	FSTL1	LCP1	PKM	SERBP1	VIM	
CLEC11A	GALNT2	LDHA	PLEC	SERPINE1	WDR1	
CLSTN1	GANAB	LDHB	PLOD1	SERPINE2	XYLT1	
CLU	GAPDH	LGALS1	PLOD2	SERPINF1	YBX1	
COL11A1	GAS6	LGALS3BP	PLOD3	SERPINH1	YWHAB	
COL12A1	GDI2	LMNA	PNP	SET	YWHAE	

Table S6. A list of proteins showing significantly increased levels in MSCs compared with osteoblasts. The protein levels were determined in the culture supernatants of *in vitro* cultured cells. The table includes (i) proteins showing detectable levels in at least three osteoblast and three MSC populations and differing significantly based on *p*-values (<0.05) and fold change (FC) ($p<0.05$) as determined by Z-score statistics; and (ii) proteins detected for ≥7 MSC and ≤1 osteoblast population. A total of 80 proteins were identified; peptides from fibronectin and periostin were annotated to two different proteins groups (isoform groups), not shown here.

PROTEIN NAME	GENE IDENTITY
Target of Nesh-SH3	ABI3BP
Disintegrin and metalloproteinase domain-containing protein 10	ADAM10
Disintegrin and metalloproteinase domain-containing protein 9	ADAM9
Adipocyte enhancer-binding protein 1	AEBP1
Fructose-bisphosphate aldolase C	ALDOC
DNA-(apurinic or apyrimidinic site) lyase;DNA-(apurinic or apyrimidinic site) lyase, mitochondrial	APEX1
Actin-related protein 2/3 complex subunit 3	ARPC3
V-type proton ATPase subunit S1	ATP6AP1
Coiled-coil domain-containing protein 80	CCDC80
CD109 antigen	CD109
Cadherin-11	CDH11
Cadherin-2	CDH2
Chitinase domain-containing protein 1	CHID1
Carbohydrate sulfotransferase 11	CHST11
C-type lectin domain family 11 member A	CLEC11A
Clusterin;Clusterin beta chain;Clusterin alpha chain	CLU
Collagen alpha-1(X) chain	COL10A1
Collagen alpha-1(XI) chain	COL11A1
Collagen alpha-1(XVIII) chain;Endostatin	COL18A1
Collagen alpha-1(I) chain	COL1A1
Collagen alpha-2(I) chain	COL1A2
Collagen alpha-1(III) chain	COL3A1
Collagen alpha-1(IV) chain;Arresten	COL4A1
Collagen alpha-2(V) chain	COL5A2
Collagen alpha-2(VI) chain	COL6A2
Collagen alpha-1(VII) chain	COL7A1
Collagen alpha-1(VIII) chain;Vastatin	COL8A1
Chondroitin sulfate proteoglycan 4	CSPG4
Collagen triple helix repeat-containing protein 1	CTHRC1
Dipeptidyl peptidase 1; Dipeptidyl peptidase 1 exclusion domain chain;Dipeptidyl peptidase 1 heavy chain;Dipeptidyl peptidase 1 light chain	CTSC
Extracellular matrix protein 1	ECM1
Eukaryotic translation initiation factor 3 subunit J	EIF3J
Eukaryotic translation initiation factor 4B	EIF4B
EMILIN-1	EMILIN1
Ectonucleotide pyrophosphatase/phosphodiesterase family member 1;Alkaline phosphodiesterase I;Nucleotide pyrophosphatase	ENPP1
Fibulin-5	FBLN5

Fibronectin	FN1
Glutamine amidotransferase-like class 1 domain-containing protein 3A, mitochondrial	GATD3A
Golgi membrane protein 1	GOLM1
Gremlin-1	GREM1
GrpE protein homolog 1, mitochondrial	GRPEL1
Hyaluronan and proteoglycan link protein 1	HAPLN1
Hemicentin-1	HMCN1
Heterogeneous nuclear ribonucleoprotein U-like protein 2	HNRNPUL2
Heat shock factor-binding protein 1	HSBP1
Stress-70 protein, mitochondrial	HSPA9
Insulin;Insulin B chain;Insulin A chain;Insulin, isoform 2	INS
Low-density lipoprotein receptor	LDLR
Lysosomal acid lipase/cholesteryl ester hydrolase	LIPA
Lysyl oxidase homolog 3	LOXL3
Latent-transforming growth factor beta-binding protein 1	LTBP1
Mannosyl-oligosaccharide 1,2-alpha-mannosidase IA	MAN1A1
Microtubule-associated protein RP/EB family member 1	MAPRE1
Cell surface glycoprotein MUC18	MCAM
Hepatocyte growth factor receptor	MET
Interstitial collagenase;22 kDa interstitial collagenase;27 kDa interstitial collagenase	MMP1
Stromelysin-3	MMP11
Major vault protein	MVP
Nidogen-1	NID1
Neuropilin-2	NRP2
Succinyl-CoA:3-ketoacid coenzyme A transferase 1, mitochondrial	OXCT1
Protocadherin gamma-C3	PCDHGC3
Procollagen C-endopeptidase enhancer 1	PCOLCE
Urokinase-type plasminogen activator	PLAU
Phospholipid transfer protein	PLTP
Periostin	POSTN
Inactive tyrosine-protein kinase 7	PTK7
60S ribosomal protein L30	RPL30
Ribosome-binding protein 1	RRBP1
Syndecan-1	SDC1
Semaphorin-7A	SEMA7A
Serpin B7	SERPINB7
Sushi repeat-containing protein SRPX	SRPX
Stanniocalcin-1	STC1
Small ubiquitin-related modifier 1	SUMO1
Serotransferrin	TF
Transforming growth factor beta-1;Latency-associated peptide	TGFB1
Tenascin	TNC
Vesicle-associated membrane protein-associated protein A	VAPA
Vasodilator-stimulated phosphoprotein	VASP

Table S7. Proteins showing increased constitutive release by *in* vitro cultured human MSCs. The table lists the proteins showing significantly increased levels in MSCs compared with osteoblasts. The table includes (i) proteins showing detectable levels in at least three osteoblast and three MSC populations and differing significantly based on *p*-values (<0.05) and fold change (FC, *p*<0.05); and (ii) proteins detected for ≥7 MSC and ≤1 osteoblast population. A total of 80 proteins were identified; peptides from fibronectin and periostin were annotated to two different proteins groups (isoform groups) not shown here. The table presents the protein identity (i.e. encoding gene), a brief description of the encoded protein and key words describing important structure/function of the protein. The information is based on the Gene database and selected references from the PubMed database. Yellow background color indicates extracellular matrix molecules, green color adhesion molecules and blue color proteases.

IDENTITY	SUMMARY	KEY WORDS
ABI3BP	*ABI family member 3 binding protein.* ABI3BP is a relatively newly identified protein whose general biological functions are not yet fully defined. It is implicated in promoting cellular senescence and cell-extracellular matrix interactions; both ABI3BP and ABI3 are downregulated in cancers further with supporting evidence that they are both important in promoting cellular senescence. ABI3BP is best known for is its role in multiple forms of cancer, acting as a tumor suppressor via promotion of cellular senescence. ABI3BP is also known as an extracellular/interstitial matrix protein and plays a role in cell-substrate adhesion (PMID 30923710).	Extracellular matrix<br>Tumor suppressor<br>Inducing senescence
ADAM9	*ADAM metallopeptidase domain 9.* This gene encodes a member of the ADAM (a disintegrin and metalloprotease domain) family. Members of this family are membrane-anchored proteins and have been implicated in cell-cell and cell-matrix interactions. The protein encoded by this gene interacts with SH3 domain-containing proteins, binds mitotic arrest deficient 2 beta protein, and is also involved in shedding of membrane-anchored heparin-binding EGF-like growth factor.	Peptidase<br>Adhesion<br>Cytokine binding
ADAM10	*ADAM metallopeptidase domain 10.* Members of the ADAM family are cell surface proteins with a unique structure possessing both potential adhesion and protease domains. This gene encodes an ADAM family member that cleaves many proteins including TNF-alpha and E-cadherin.	Peptidase<br>Adhesion
AEBP1	*AE binding protein 1.* This member of the carboxypeptidase A protein family may function as a transcriptional repressor.	Peptidase<br>Transcription
ALDOC	*Aldolase C, fructose-bisphosphate.* This is a member of the aldolase family of enzymes that is an enzyme of the glycolysis pathway and catalyzes the conversion of fructose-1,6-bisphosphate to glyceraldehyde-3-phosphate and dihydroxyacetone phosphate.	Metabolism<br>Glycolysis
APEX1	*Apurinic/apyrimidinic endodeoxyribonuclease 1.* The APEX gene encodes the major AP endonuclease in human cells. It encodes the APEX endonuclease, a DNA repair enzyme with apurinic/apyrimidinic (AP) activity. Such AP activity sites occur frequently in DNA molecules by spontaneous hydrolysis, by DNA damaging agents or by DNA glycosylases that remove specific abnormal bases.	Endonuclease<br>DNA repair
ARPC3	*Actin related protein 2/3 complex subunit 3.* This protein is one of seven subunits of the human Arp2/3 protein complex that is implicated in the control of actin polymerization in cells.	Cytoskeleton<br>Actin
ATP6AP1	*ATPase H+ transporting accessory protein 1.* This is a component of a multisubunit enzyme that mediates acidification of eukaryotic intracellular organelles, i.e. the Vacuolar ATPase (V-ATPase) that is comprised of a cytosolic V1 (site of the ATP	V-ATPase<br>Protein sorting

	catalytic site) and a transmembrane V0 domain. V-ATPase dependent organelle acidification is necessary for such intracellular processes as protein sorting and receptor-mediated endocytosis.	Endocytosis
CCDC80	*Coiled-coil domain containing 80.* Involved in FAK/E-cadherin signaling (PMID 30214608). Focal adhesion kinase (FAK) is a non-receptor tyrosine kinase involved in the development and progression of cancer.	Adhesion<br>Cadherin
CD109	*CD109.* This glycosyl phosphatidylinositol (GPI)-linked glycoprotein localizes to the surface. It binds to and negatively regulates signaling by transforming growth factor beta (TGF-beta).	Cell surface<br>TGFβ
CDH11	*Cadherin 11.* This gene encodes a type II classical cadherin from the cadherin superfamily, integral membrane proteins that mediate calcium-dependent cell-cell adhesion. Type II (atypical) cadherins are defined based on their lack of a HAV cell adhesion recognition sequence specific to type I cadherins.	Cadherin
CDH2	*Cadherin 2.* This classical cadherin has multiple transcript variants, at least one of which encodes a preproprotein that is proteolytically processed to generate a calcium-dependent cell adhesion molecule and glycoprotein.	Adhesion
CHID1	*Chitinase domain containing 1.* This chitinase-like molecule may have a role as a growth factor. It interacts with syndecan, stimulated chemokine production and MMP9 release.	Syndecan<br>Chemokine, MMP9.
CHST11	*Carbohydrate sulfotransferase 11.* The protein belongs to the sulfotransferase 2 family. It is localized to the Golgi membrane, and catalyzes the transfer of sulfate to position 4 of the N-acetylgalactosamine (GalNAc) residue of chondroitin. Chondroitin sulfate constitutes the predominant proteoglycan and is distributed on the surfaces of many cells and extracellular matrices.	Golgi<br>Cell surface
CLEC11A	*C-type lectin domain containing 11A.* This protein belongs to the C-type lectin superfamily. It is a secreted sulfated glycoprotein and functions as a growth factor for primitive hematopoietic progenitor cells.	Hematopoietic growth factor
CLU	Clusterin. The protein is a secreted chaperone that can under some stress conditions also be found in the cell cytosol. It is possibly involved in several basic biological events such as cell death and cancer progression.	Chaperone
COL10A1	*Collagen type X alpha 1 chain.* The alpha chain of type X collagen is a short chain collagen. Unlike type VIII collagen, the other short chain collagen, type X collagen is a homotrimer.	Collagen<br>Extracellular matrix
COL11A1	*Collagen type XI alpha 1 chain.* This gene encodes one of the two alpha chains of type XI collagen, a minor fibrillar collagen. Type XI collagen is a heterotrimer but the third alpha chain is a post-translationally modified alpha 1 type II chain.	Collagen<br>Extracellular matrix
COL18A1	*Collagen type XVIII alpha 1 chain.* This gene encodes the alpha chain of type XVIII collagen. This collagen is one of the multiplexins, extracellular matrix proteins that contain multiple triple-helix domains (collagenous domains) interrupted by non-collagenous domains. Proteolytic processing at several endogenous cleavage sites in the C-terminal domain results in production of endostatin, a potent antiangiogenic protein that is able to inhibit angiogenesis and tumor growth.	Collagen<br>Extracellular matrix<br>Endostatin<br>Antiangiogenic
COL1A1	*Collagen type I alpha 1 chain.* This gene encodes the pro-alpha1 chains of type I collagen whose triple helix comprises two alpha1 chains and one alpha2 chain. Type I is a fibril-forming collagen.	Collagen<br>Extracellular matrix
COL1A2	*Collagen type III alpha 2 chain.* This gene encodes the pro-alpha2 chain of type I collagen whose triple helix comprises two alpha1 chains and one alpha2 chain.	Collagen<br>Extracellular matrix
COL3A1	*Collagen type III alpha 1 chain.* This gene encodes the pro-alpha1 chains of type III collagen, a fibrillar collagen.	Collagen<br>Extracellular matrix
COL4A1	*Collagen type IV alpha 1 chain.* This gene encodes a type IV collagen alpha protein. Type IV collagen proteins are integral components of basement membranes. The protein consists of an amino-terminal 7S domain, a triple-helix forming collagenous domain, and a carboxy-terminal non-collagenous domain. It functions as part of a heterotrimer and interacts with other extracellular matrix components such as perlecans, proteoglycans, and laminins. In addition, proteolytic cleavage of the	Collagen<br>Extracellular matrix<br>Arresten<br>Antiangiogenic

	non-collagenous carboxy-terminal domain results in a biologically active fragment known as arresten, which has anti-angiogenic and tumor suppressor properties.	Tumor suppressor
COL5A2	*Collagen type V alpha 2 chain.* This protein is the alpha chain for one of the low abundance fibrillar collagens. Fibrillar collagen molecules are trimers that can be composed of one or more types of alpha chains. Type V collagen is found in tissues containing type I collagen and appears to regulate the assembly of heterotypic fibers composed of both type I and type V collagen. This gene product is closely related to type XI collagen and it is possible that the collagen chains of types V and XI constitute a single collagen type with tissue-specific chain combinations.	Extracellular matrix<br>Collagen
COL6A2	*Collagen type VI alpha 2 chain.* This gene encodes one of the three alpha chains of type VI collagen, a beaded filament collagen found in most connective tissues. The product of this gene contains several domains similar to von Willebrand Factor type A domains. These domains have been shown to bind extracellular matrix proteins, an interaction that explains the importance of this collagen in organizing matrix components.	Collagen<br>Extracellular matrix
COL7A1	*Collagen type VII alpha 1 chain.* This gene encodes the alpha chain of type VII collagen. The type VII collagen fibril, composed of three identical alpha collagen chains, functions as an anchoring fibril.	Collagen<br>Extracellular matrix
COL8A1	*Collagen type VII alpha 1 chain.* This gene encodes one of the two alpha chains of type VIII collagen. The gene product is a short chain collagen. The type VIII collagen fibril can be either a homo- or a heterotrimer.	Collagen<br>Extracellular matrix
CSPG4	*Chondroitin sulfate proteoglycan 4.* This chondroitin sulfate proteoglycan plays a role in stabilizing cell-substratum interactions. CSPG4 represents an integral membrane chondroitin sulfate proteoglycan.	Adhesion<br>Extracellular matrix
CTHRC1	*Collagen triple helix repeat containing 1.* This protein that is involved in vascular remodeling.	Angiogenesis?
CTSC	*Cathepsin C.* This protein is a member of the peptidase C1 family and is a lysosomal cysteine proteinase that appears to be a central coordinator for activation of many serine proteinases. Alternative splicing results in multiple transcript variants, at least one of which encodes a preproprotein that is proteolytically processed to generate heavy and light chains forming a disulfide-linked dimer. A portion of the propeptide acts as an intramolecular chaperone for the folding and stabilization of the mature enzyme.	Peptidase<br>Lysosome<br>Chaperone
ECM1	*Extracellular matrix protein 1.* This gene encodes a soluble protein that is involved in angiogenesis. It also interacts with a variety of extracellular and structural proteins.	Extracellular matrix<br>Angiogenesis
EIF3J	*Eukaryotic translation initiation factor 3 subunit J.* This gene encodes a core subunit of the eukaryotic initiation factor 3 complex, which participates in the initiation of translation by aiding in the recruitment of protein and mRNA components to the 40S ribosome.	Ribosome
EIF4B	*Eukaryotic translation initiation factor 4B.* Three proteins specifically required for the translation initiation of natural mRNAs, i.e. eIF4A, eIF4B, and eIF4F. The regulation of translation exhibited through the mammalian target of rapamycin (mTOR) pathway is predominately seen as the phosphorylation of 4E-BP, an inhibitor of protein synthesis that functions by binding to the cap binding subunit of eIF4F (eIF4E) (PMID 26324716)	Protein synthesis
EMILIN1	*Elastin microfibril interfacer 1.* This extracellular matrix glycoprotein that is characterized by an N-terminal microfibril interface domain, a coiled-coiled alpha-helical domain, a collagenous domain and a C-terminal globular C1q domain. It associates with elastic fibers at the interface between elastin and microfibrils.	Extracellular matrix
ENPP1	*Ectonucleotide pyrophosphatase/phosphodiesterase 1.* This pyrophosphatase/phosphodiesterase (ENPP) family member is a type II transmembrane glycoprotein comprising two identical disulfide-bonded subunits. It has broad specificity and cleaves a variety of substrates, including phosphodiester bonds of nucleotides and nucleotide sugars and pyrophosphate bonds of nucleotides and nucleotide sugars. It may also be important for insulin effects.	Phosphodiesterase<br>Transmembrane<br>Insulin?

FBLN5	*Fibulin 5.* This gene encodes a secreted, extracellular matrix protein containing an Arg-Gly-Asp (RGD) motif and calcium-binding EGF-like domains. It promotes adhesion of endothelial cells through interaction of integrins and the RGD motif. It may play a role in vascular development and remodeling.	Extracellular matrix<br>Vascular development
FN1	*Fibronectin 1.* This glycoprotein is present in a soluble dimeric form in plasma, and in a dimeric or multimeric form at the cell surface and in extracellular matrix. The encoded preproprotein is proteolytically processed to generate the mature protein. Fibronectin is involved in cell adhesion and migration. The gene has three regions subject to alternative splicing, with the potential to produce 20 different transcript variants, at least one of which encodes an isoform that undergoes proteolytic processing.	Extracellular matrix<br>Adhesion
GATD3A	*Glutamine amidotransferase like class 1 domain containing 3A.* This is a potential mitochondrial protein that is a member of the DJ-1/PfpI gene family.	Mitochondria<br>Metabolism
GOLM1	*Golgi membrane protein 1.* The Golgi complex plays a key role in the sorting and modification of proteins exported from the endoplasmic reticulum. This protein is a type II Golgi transmembrane protein. It processes proteins synthesized in the rough endoplasmic reticulum and assists in the transport of protein cargo through the Golgi apparatus.	Golgi<br>Protein transport
GREM1	*Gremlin 1, DAN family BMP antagonist.* This gene encodes a member of the BMP (bone morphogenic protein) antagonist family. The CAN (cerberus and dan) subfamily of BMP antagonists, to which this gene belongs, is characterized by a C-terminal cystine knot with an eight-membered ring. The antagonistic effect of the secreted glycosylated protein encoded by this gene is likely due to its direct binding to BMP proteins.	BMP antagonist
GRPEL1	*GrpE like 1, mitochondrial.* The mitochondrial heat shock protein 70 (mtHsp70) is a chaperone contributing to mitochondrial protein quality control. The two nuclear exchange factors GrpE-like 1 (GrpEL1) and GrpEL2 modulate mtHsp70's function and associate with mtHsp70 as a hetero-oligomeric subcomplex and regulate mtHsp70 function (PMID 28848044).	Mitochondria<br>HSP70<br>Chaperone
HAPLN1	*Hyaluronan and proteoglycan link protein 1.* The three ECM components hyaluronan and proteoglycan link protein 1 (HAPLN1), lumican and collagen I (collectively, HLC) seem to be form an interacting complex (PMID 30078576)	Extracellular matrix<br>Collagen
HMCN1	*Hemicentin 1.* This large extracellular member of the immunoglobulin superfamily forms long tracks at specific extracellular sites that are involved in many processes, including cellular anchorage and organization of hemidesmosomes	Extracellular matrix<br>Adhesion, desmosomes
HNRNPUL2	*Heterogeneous nuclear ribonucleoprotein U like 2.* Long noncoding RNAs (lncRNAs) can be important for development and chemosensitivity of certain cancers. It can form a complex with heterogeneous nuclear ribonucleoprotein U-like 2 protein (hnRNPUL2) and direct the transport of hnRNPUL2 between the nucleus and cytoplasm. hnRNPUL2 may then accumulated in the cytoplasm and increase the RNA stability (PMID 28594403).	Noncoding RNA
HSBP1	*Heat shock factor binding protein 1.* Heat shock factor binding protein 1 (HSBP1), is a 76-amino-acid protein that binds to heat shock factor 1(HSF1), which is a transcription factor involved in the heat shock response. During HS response, HSF1 undergoes conformational transition from an inert non-DNA-binding monomer to active functional trimers. HSBP1 is nuclear-localized and interacts with the active trimeric state of HSF1 to negatively regulate HSF1 DNA-binding activity. Overexpression of HSBP1 in mammalian cells represses the transactivation activity of HSF1.	Heat shock response<br>Transcription
HSPA9	*Heat shock protein family A (Hsp70) member 9.* This gene encodes a member of the heat shock protein 70 gene family. The encoded protein is primarily localized to the mitochondria but is also found in the endoplasmic reticulum, plasma membrane and cytoplasmic vesicles. This protein is a heat-shock cognate protein. This protein plays a role in cell proliferation, stress response and maintenance of the mitochondria.	Heat shock response<br>Mitochondria<br>Endoplasmic reticulum
INS	*Insulin.* Insuling is an important regulator of A cellular metabolism and proliferation.	Metabolism

LDLR	*Low density lipoprotein receptor.* The low density lipoprotein receptor (LDLR) gene family consists of cell surface proteins involved in receptor-mediated endocytosis of specific ligands. Low density lipoprotein (LDL) is normally bound at the cell membrane and taken into the cell ending up in lysosomes where the protein is degraded and the cholesterol is made available for repression of microsomal enzyme 3-hydroxy-3-methylglutaryl coenzyme A (HMG CoA) reductase, the rate-limiting step in cholesterol synthesis. At the same time, a reciprocal stimulation of cholesterol ester synthesis takes place.	Cholesterol metabolism<br>Cell surface<br>Lysosome
LIPA	*Lipase A, lysosomal acid type.* This gene encodes lipase A, the lysosomal acid lipase that functions in the lysosome to catalyze the hydrolysis of cholesteryl esters and triglycerides.	Cholesterol metabolism<br>Lysosome
LOXL3	*Lysyl oxidase like 3.* This lysyl oxidase likely functions as an amine oxidase and plays a role in the formation of crosslinks in collagens and elastin.	Extracellular matrix<br>Lysyl oxidase
LTBP1	*Latent transforming growth factor beta binding protein 1.* The protein belongs to the family of latent TGF-beta binding proteins (LTBPs). The secretion and activation of TGF-beta is regulated by their association with latency-associated proteins and with latent TGF-beta binding proteins. This protein targets latent complexes of transforming growth factor beta to the extracellular matrix, where the latent cytokine is subsequently activated by several different mechanisms.	TGFβ
MAN1A1	*Mannosidase alpha class 1A member 1.* This class I mammalian Golgi 1,2-mannosidase member is a type II transmembrane protein. It catalyzes the hydrolysis of three terminal mannose residues from peptide-bound Man(9)-GlcNAc(2) oligosaccharides and belongs to family 47 of glycosyl hydrolases.	Mannosidase
MAPRE1	*Microtubule associated protein RP/EB family member 1.* The protein localizes to microtubules, especially the growing ends, in interphase cells. During mitosis, the protein is associated with the centrosomes and spindle microtubules. The protein also associates with components of the dynactin complex and the intermediate chain of cytoplasmic dynein. Because of these associations, it is thought that this protein is involved in the regulation of microtubule structures and chromosome stability.	Cytoskeleton<br>Microtubule<br>Centrosome<br>Mitosis
MCAM	*Melanoma cell adhesion molecule.* The molecule can mediate cell-cell adhesion (PMID 25595133).	Adhesion
MET	*MET proto-oncogene, receptor tyrosine kinase.* This gene encodes a member of the receptor tyrosine kinase family of proteins and the product of the proto-oncogene MET. The encoded preproprotein is proteolytically processed to generate alpha and beta subunits that are linked via disulfide bonds to form the mature receptor. Binding of its ligand, hepatocyte growth factor, induces dimerization and activation of the receptor, which plays a role in cellular survival.	Oncogene<br>HGF
MMP1	*Matrix metallopeptidase 1.* This member of the peptidase M10 family of matrix metalloproteinases (MMPs); these proteins are involved in the breakdown of extracellular matrix in normal physiological processes. The encoded preproprotein is proteolytically processed to generate the mature protease. This secreted protease breaks down the interstitial collagens, including types I, II, and III.	Extracellular protease<br>Collagen
MMP11	*Matrix metallopeptidase 11.* Proteins of the matrix metalloproteinase (MMP) family are involved in the breakdown of extracellular matrix. Most MMPs are secreted as inactive proproteins which are activated when cleaved by extracellular proteinases. However, the enzyme encoded by this gene is activated intracellularly by furin within the constitutive secretory pathway. Also in contrast to other MMPs, this enzyme cleaves alpha 1-proteinase inhibitor but weakly degrades structural proteins of the extracellular matrix.	Extracellular protease
MVP	*Major vault protein.* This protein is the major component of the vault complex. Vaults are multi-subunit ribonucleoprotein structures that may be involved in nucleo-cytoplasmic transport. The encoded protein may play a role in multiple cellular processes by regulating the MAP kinase, JAK/STAT and phosphoinositide 3-kinase/Akt signaling pathways. The encoded protein also plays a role in multidrug resistance.	Nucleo-cytoplasmic transport<br>Intracellular signaling

NID1	*Nidogen 1.* This member of the nidogen family of basement membrane glycoproteins interacts with several other components of basement membranes, and may play a role in cell interactions with the extracellular matrix.	Extracellular matrix
NRP2	*Neurophilin 2.* This member of the neuropilin family of receptor proteins is a transmembrane protein that binds to SEMA3C protein {sema domain, immunoglobulin domain (Ig), short basic domain, secreted, (semaphorin) 3C} and SEMA3F protein {sema domain, immunoglobulin domain (Ig), short basic domain, secreted, (semaphorin) 3F}. It interacts with vascular endothelial growth factor (VEGF). The protein may have a role in angiogenesis.	Semaphorin<br>VEGF<br>Membrane molecule<br>Angiogenesis?
OXCT1	*3-oxoacid CoA-transferase 1.* This member of the 3-oxoacid CoA-transferase gene family is a homodimeric mitochondrial matrix enzyme that plays a role in ketone body catabolism.	Mitochondria<br>Metabolism
PCDHGC3	*Protocadherin gamma subfamily C, 3.* This member of the protocadherin gamma gene cluster has an immunoglobulin-like organization. The gamma gene cluster includes 22 genes divided into 3 subfamilies. Subfamily C contains 3 genes. Each variable region exon encodes the extracellular region, which includes 6 cadherin ectodomains and a transmembrane region. The constant region exons encode the common cytoplasmic region. These neural cadherin-like cell adhesion proteins most likely play a critical role in the establishment and function of specific cell-cell connections.	Adhesion Cell-cell contact
PCOLCE	*Procollagen C-endopeptidase enhancer.* Fibrillar collagen types I-III are synthesized as precursor molecules known as procollagens. These precursors contain amino- and carboxyl-terminal peptide extensions known as N- and C-propeptides, respectively, which are cleaved, upon secretion of procollagen from the cell, to yield the mature triple helical, highly structured fibrils. This encoded glycoprotein binds and drives the enzymatic cleavage of type I procollagen and heightens C-proteinase activity.	Extracellular matrix<br>Peptidase<br>Collagen
PLAU	*Plasminogen activator, urokinase.* This secreted serine protease converts plasminogen to plasmin.	Coagulation/protease
PLTP	*Phspholipid transfer protein.* The protein is one of at least two lipid transfer proteins found in human plasma. The encoded protein transfers phospholipids from triglyceride-rich lipoproteins to high density lipoprotein (HDL). In addition to regulating the size of HDL particles, this protein may be involved in cholesterol metabolism.	Lipid metabolism<br>Cholesterol metabolism?
POSTN	*Periostin.* This secreted extracellular matrix protein binds to integrins to support adhesion and migration of cells. It plays a role in cancer stem cell maintenance and metastasis.	Extracellular matrix<br>Integrin<br>Stem cell maintenence
PTK7	*Protein tyrosine kinase 7.* This receptor protein tyrosine kinase lacks detectable catalytic tyrosine kinase activity, is involved in the Wnt signaling pathway and plays a role in multiple cellular processes including polarity and adhesion.	Tyrosine kinase<br>Wnt signaling<br>Adhesion
RPL30	*Ribosomal protein L30.* This ribosomal protein is a component of the 60S subunit. The protein belongs to the L30E family of ribosomal proteins. It is located in the cytoplasm. This gene is co-transcribed with the U72 small nucleolar RNA gene, which is located in its fourth intron.	Ribosome
RRBP1	*Ribosome binding protein 1.* This gene encodes a ribosome-binding protein of the endoplasmic reticulum (ER) membrane. Studies suggest that this gene plays a role in ER proliferation, secretory pathways and secretory cell differentiation, and mediation of ER-microtubule interactions.	Ribosome<br>Endoplasmic reticulum<br>Secretion
SDC1	*Syndecan 1.* This transmembrane (type I) heparan sulfate proteoglycan is a member of the syndecan proteoglycan family. The syndecans mediate cell binding, cell signaling, and cytoskeletal organization. The syndecan-1 protein functions as an integral membrane protein and participates in cell proliferation, cell migration and cell-matrix interactions via its receptor for extracellular matrix proteins.	Cell surface<br>Extracellular matrix<br>Adhesion

SEMA7A	*Semaphorin 7A.* This preproprotein is proteolytically processed to generate the mature glycosylphosphatidylinositol (GPI)-anchored membrane glycoprotein.	Membrane protein
SERPINB7	*Serpin family B member 7.* This gene encodes a member of a family of proteins which function as protease inhibitors.	Protease inhibition
SRPX	*Sushi repeat containing protein X-linked.* The protein is possibly a perioxiredoxin (PMID 20964819).	Peroxiredoxin
STC1	*Stanniocalcin 1.* This gene encodes a secreted, homodimeric glycoprotein. It is phosphorylated by protein kinase C exclusively on its serine residues. The protein may play a role in the regulation of calcium and phosphate transport, cell metabolism, or cellular calcium/phosphate homeostasis.	Metabolism<br>Calcium/phosphate homeostasis
SUMO1	*Small ubiquitin like modifier 1.* This protein is a member of the SUMO (small ubiquitin-like modifier) family and functions in a manner similar to ubiquitin in that it is bound to target proteins as part of a post-translational modification system. However, unlike ubiquitin which targets proteins for degradation, this protein is involved in a variety of cellular processes, such as nuclear transport, transcriptional regulation, apoptosis, and protein stability. It is not active until the last four amino acids have been cleaved off.	Posttranscriptional modification
TF	*Transferrin.* This glycoprotein transports iron from the intestine, reticuloendothelial system, and liver parenchymal cells to all proliferating cells in the body. This protein may also have a physiologic role as granulocyte/pollen-binding protein (GPBP).	Iron<br>Neutrophil
TGFB1	*Transforming growth factor beta 1.* This gene encodes a secreted ligand of the TGF-beta (transforming growth factor-beta) superfamily of proteins. Ligands of this family bind various TGF-beta receptors leading to recruitment and activation of SMAD family transcription factors that regulate gene expression. The encoded preproprotein is proteolytically processed to generate a latency-associated peptide (LAP) and a mature peptide, and is found in either a latent form composed of a mature peptide homodimer, a LAP homodimer, and a latent TGF-beta binding protein, or in an active form consisting solely of the mature peptide homodimer. The mature peptide may also form heterodimers with other TGFB family members. This encoded protein regulates cell proliferation, differentiation and growth, and can modulate expression and activation of other growth factors including interferon gamma and tumor necrosis factor alpha.	TGF<br>TNF<br>IFN<br>Cell proliferation
TNC	*Tenacin C.* This extracellular matrix protein that contains multiple EGF-like and fibronectin type-III domains. It is implicated in migration.	Extracellular matrix<br>Migration
VAPA	*VAMP associated protein A.* The protein is a type IV membrane protein that is present in the plasma membrane and intracellular vesicles. It may also be associated with the cytoskeleton. This protein may function in vesicle trafficking, membrane fusion, protein complex assembly and cell motility.	Membrane protein<br>Intracellular trafficking<br>Cytoskeleton<br>Secretion
VASP	*Vasodilator stimulated phosphoprotein.* This is a member of the Ena-VASP protein family that targets to focal adhesions. Their C-terminal EVH2 domain mediates tetramerization and binds both G and F actin. VASP is associated with filamentous actin formation and likely plays a widespread role in cell adhesion and motility. VASP may also be involved in the intracellular signaling pathways that regulate integrin-extracellular matrix interactions.	Adhesion<br>Actin<br>Integrin-extracellular matrix

Table S8. A list of proteins showing significantly increased levels in osteoblasts compared with MSCs. The protein levels were determined in the culture supernatants of *in vitro* cultured cells. The table includes (i) proteins showing detectable levels in at least three osteoblast and three MSC populations and differing significantly based on *p*-values (<0.05) and Fold Change (FC, $p<0.05$); and (ii) proteins detected for ≥7 osteoblast and ≤1 MSC populations. A total of 36 proteins were identified.

PROTEIN NAME	GENE ID
Aggrecan core protein;Aggrecan core protein 2	ACAN
Adenosylhomocysteinase	AHCY
Apolipoprotein C-III	APOC3
Apoptosis regulator BAX	BAX
Complement C3	C3
Protein canopy homolog 2	CNPY2
Collagen alpha-1(II) chain;Collagen alpha-1(II) chain;Chondrocalcin	COL2A1
Lysosomal protective protein	CTSA
Cathepsin D;Cathepsin D light chain;Cathepsin D heavy chain	CTSD
EGF-containing fibulin-like extracellular matrix protein 1	EFEMP1
Elastin	ELN
Coagulation factor X	F10
Hemoglobin subunit alpha	HBA1
Heat shock 70 kDa protein 6;Putative heat shock 70 kDa protein 7	HSPA6;HSPA7
Insulin-like growth factor-binding protein complex acid labile subunit	IGFALS
Insulin-like growth factor-binding protein 3	IGFBP3
Laminin subunit beta-2	LAMB2
Galectin-3-binding protein	LGALS3BP
Metallothionein-2	MT2A
Matrix-remodeling-associated protein 5	MXRA5
Myosin-14	MYH14
Neuroblastoma suppressor of tumorigenicity 1	NBL1
Proenkephalin-A	PENK
Podocan	PODN
Proteasome subunit beta type-8	PSMB8
Parathymosin	PTMS
Ras-related protein Rab-7a	RAB7A
CapZ-interacting protein	RCSD1
Signal peptide, CUB and EGF-like domain-containing protein 3	SCUBE3
Syntenin-1	SDCBP
Selenoprotein P	SEPP1
Plasma protease C1 inhibitor	SERPING1
Small ubiquitin-related modifier 2	SUMO2
Thrombospondin-4	THBS4
Testis-specific serine/threonine-protein kinase 4	TSSK4
Pantetheinase	VNN1

Table S9. Proteins showing increased constitutive release by *in* vitro cultured human osteoblasts. The table lists the proteins showing significantly increased levels in osteoblasts compared with MSCs. The table includes (i) proteins showing detectable levels in at least three osteoblast and three MSC populations and differing significantly based on *p*-values (<0.05) and Fold Change (FC, *p*<0.05); and (ii) proteins detected for ≥7 osteoblast and ≤1 MSC populations. We identified 36 peptides derived from 36 proteins. The table presents the protein identity (i.e. encoding gene), a brief description of the encoded protein and key words describing important structure/function of the protein. The information is based on the Gene database and selected references from the PubMed database. Yellow background color indicates extracellular matrix molecules, green color adhesion molecules and blue color proteases.

IDENTITY	SUMMARY	KEY WORDS
ACAN	*Aggrecan.* This protein is a member of the aggrecan/versican proteoglycan family and a part of the extracellular matrix.	Extracellular matrix
AHCY	*Adenosylhomocysteinase.* S-adenosylhomocysteine hydrolase belongs to the adenosylhomocysteinase family and catalyzes the reversible hydrolysis of S-adenosylhomocysteine (AdoHcy) to adenosine (Ado) and L-homocysteine (Hcy). Thus, it regulates the intracellular S-adenosylhomocysteine (SAH) concentration thought to be important for transmethylation reactions.	Metabolism<br>Homocystein<br>Adenosin
APOC3	*Apolipoprotein C3.* This protein is a component of triglyceride (TG)-rich lipoproteins (TRLs) including very low density lipoproteins (VLDL), high density lipoproteins (HDL) and chylomicrons. The encoded protein plays a role in role in the metabolism of these TRLs through multiple modes.	Lipid metabolism
BAX	*BCL2 associated X, apoptosis regulator.* This BCL2 family protein and forms a heterodimer with BCL2; it thereby functions as an apoptotic activator. It interacts with, and increase the opening of, the mitochondrial voltage-dependent anion channel (VDAC), which leads to the loss in membrane potential and the release of cytochrome c. The expression of this gene is regulated by the tumor suppressor p53 and has been shown to be involved in p53-mediated apoptosis.	Proapoptotic<br>p53
C3	*Complement factor C3.* Complement component C3 plays a central role in the activation of complement system. Its activation is required for both classical and alternative complement activation pathways. The encoded preproprotein is proteolytically processed to generate alpha and beta subunits that form the mature protein. The C3a peptide modulates inflammation	Complement<br>Inflammation
CNPY2	*Canopy FGF signaling regulator 2.*	Cytokine signaling
COL2A1	*Collagen type II alpha 1 chain.* This gene encodes the alpha-1 chain of type II collagen, a fibrillar collagen found.	Extracellular matrix
CTSA	*Cathepsin A.* This member of the peptidase S10 family of serine carboxypeptidases. This enzyme possesses deamidase, esterase and carboxypeptidase activities and acts as a scaffold in the lysosomal multienzyme complex.	Peptidase, lysosome
CTSD	*Cathepsin D.* This gene encodes a member of the A1 family of peptidases. The encoded preproprotein is proteolytically processed to generate cathepsin D light and heavy chains, which heterodimerize to form the mature enzyme. This enzyme exhibits pepsin-like activity.	Peptidase
EFEMP1	*EGF containing fibulin extracellular matrix protein 1.* This protein is a member of the fibulin family of extracellular matrix glycoproteins. This gene is upregulated in malignant gliomas and may play a role in the aggressive nature of these tumors.	Extracellular matrix<br>Cancer
ELN	*Elastin.* This encoded protein is one of the two components of elastic fibers. Elastic fibers comprise part of the extracellular matrix. Degradation products of the encoded protein, known as elastin-derived peptides or elastokines, bind the elastin receptor complex and other receptors and stimulate migration and proliferation and can also contribute to cancer progression.	Extracellular matrix

F10	*Coagulation factor X.* This gene encodes the vitamin K-dependent coagulation factor X of the blood coagulation cascade.	Coagulation
HBA1	*Hemoglobin subunit alpha 1.* Two alpha chains plus two beta chains constitute HbA, which in normal adult life comprises about 97% of the total hemoglobin; alpha chains combine with delta chains to constitute HbA-2, which with HbF (fetal hemoglobin) makes up the remaining 3% of adult hemoglobin.	Erythrocyte
HSPA6	*Heat shock protein family A (Hsp70) member 6.*	Chaperone
IGFALS	*Insulin like growth factor binding protein acid labile subunit.* This serum protein binds insulin-like growth factors, increasing their half-life and their vascular localization.	IGF<br>Plasma protein
IGFBP3	*Insulin like growth factor binding protein 3.* This insulin-like growth factor binding protein (IGFBP) forms a complex with the insulin-like growth factor acid-labile subunit (IGFALS) and either insulin-like growth factor (IGF) I or II. In this form, it circulates in the plasma, prolonging the half-life of IGFs and altering their interaction with cell surface receptors.	IGF<br>Plasma protein
LAMB2	*Laminin subunit beta 2.* Laminins are a family of extracellular matrix glycoproteins, and are the major noncollagenous constituent of basement membranes. Laminins, composed of 3 non identical chains: laminin alpha, beta and gamma (formerly A, B1, and B2, respectively), form a cruciform structure consisting of 3 short arms, each formed by a different chain, and a long arm composed of all 3 chains. Different alpha, beta and gamma chain isomers combine to give rise to different heterotrimeric laminin isoforms which are designated by Arabic numerals. This gene encodes the beta chain isoform laminin, beta 2. The beta 2 chain contains the 7 structural domains typical of beta chains of laminin, including the short alpha region.	Extracellular matrix
LGALS3BP	*Galectin 3 binding protein.* The galectins are a family of beta-galactoside-binding proteins implicated in modulating cell-cell and cell-matrix interactions. LGALS3BP has been found elevated in the serum of patients with cancer. It appears to be implicated in immune responses associated with natural killer and lymphokine-activated killer cell cytotoxicity. The native protein binds specifically to a human macrophage-associated lectin known as Mac-2 and also binds galectin 1.	Extracellular matrix<br>Cell adhesion<br>Monocyte (Mac-1)
MT2A;MT1X;<br>MT1G;MT1M	*Metallothioenin 2A,* This gene is a member of the metallothionein family of genes; these proteins bind divalent heavy metal ions and alter intracellular concentration of heavy metals. These proteins act as anti-oxidants, protect against hydroxyl free radicals, are important in homeostatic control of metal in the cell, and play a role in detoxification of heavy metals. The encoded protein interacts with the protein encoded by the homeobox containing 1 gene in some cell types, controlling intracellular zinc levels, affecting apoptotic and autophagy pathways.	Anti-oxidant<br>Apoptosis
MXRA5	*Matrix remodeling associated 5.* This matrix-remodelling associated protein contains 7 leucine-rich repeats and 12 immunoglobulin-like C2-type domains related to perlecan.	Extracellular matrix
MYH14	*Myosin heavy chain 14.* This gene encodes a member of the myosin superfamily. The protein represents a conventional non-muscle myosin. Myosins are actin-dependent motor proteins with diverse functions including regulation of cytokinesis, cell motility, and cell polarity.	Cytoskeleton<br>Actin
NBL1	*NBL1, DAN family BMP antagonist.* This protein is the founding member of the evolutionarily conserved CAN (Cerberus and DAN) family of proteins. These proteins are secreted, and act as BMP (bone morphogenetic protein) antagonists by binding to BMPs and preventing them from interacting with their receptors.	BMP antagonist<br>Extracellular
PENK	*Preproenkephalin.* This preproprotein is proteolytically processed to generate multiple protein products.	Extracellular
PODN	*Podocan.* The protein is a member of the small leucine-rich repeat protein family and contains an amino terminal CX3CXCX7C cysteine-rich cluster followed by a leucine-rich repeat domain. Studies suggest that this protein could function to inhibit proliferation and migration. Podocan regulates Wnt/β-catenin through Wnt4 to promote MDSC differentiation (PMID 31447699).	Migration?<br>Wnt/β-catenin

PSMB8	*Proteasome 20S subunit beta 8.* The proteasome is a multicatalytic proteinase complex. This protein is a member of the proteasome B-type family, also known as the T1B family. Proteolytic processing is required to generate a mature subunit.	Proteasome
PTMS	*Parathymosin.* It is possibly a regulator of transcription and proliferation (PMID 29541218).	Transcription?
RAB7A	*RAB7A, member RAS oncogene family.* RAB family members are small, RAS-related GTP-binding proteins that are important regulators of vesicular transport. Each RAB protein targets multiple proteins that act in exocytic / endocytic pathways. This protein regulates vesicle traffic in the late endosomes and also from late endosomes to lysosomes.	GTB binding<br>Vesicular transport<br>Exocytosis/endocytosis
RCSD1	RCSD domain containing 1. The protein is a regulator of Wnt signaling.	Intracellular signaling
SCUBE3	*Signal peptide, CUB domain and EGF like domain containing 3.* This signal peptide is upregulated in lung cancer tumor tissue compared to healthy tissue and is associated with loss of the epithelial marker E-cadherin and with increased expression of vimentin. It is also a transforming growth factor beta receptor ligand. Both the full length protein and C-terminal fragment can bind to the transforming growth factor beta type II receptor.	Cadherin<br>Vimentin<br>TGFβ
SDCBP	*Syndecan binding protein.* The protein was initially identified as a molecule linking syndecan-mediated signaling to the cytoskeleton. It may also affect cytoskeletal-membrane organization, cell adhesion, protein trafficking, and the activation of transcription factors. The protein is primarily localized to membrane-associated adherens junctions and focal adhesions but is also found at the endoplasmic reticulum and nucleus.	Cytoskeleton<br>Adhesion<br>Intracellular transport<br>Transcription?
SEPP1	*Selenoprotein P.* A selenoprotein that is predominantly secreted into the plasma. It is unique in that it contains multiple selenocysteine (Sec) residues per polypeptide (10 in human), and accounts for most of the selenium in plasma. It has been implicated as an extracellular antioxidant, and in the transport of selenium to extra-hepatic tissues via apolipoprotein E receptor-2 (apoER2).	Extracellular<br>Transport<br>Antioxidant
SERPING1	*Serpin family G member 1.* This highly glycosylated plasma protein is involved in the regulation of the complement cascade. Its encoded protein, C1 inhibitor, inhibits activated C1r and C1s of the first complement component and thus regulates complement activation.	Complement inhibition
SUMO2	*Small ubiquitin like modifier 2.* This protein is a member of the SUMO (small ubiquitin-like modifier) protein family. It is bound to target proteins as part of a post-translational modification system. However, unlike ubiquitin which targets proteins for degradation, this protein is involved in a variety of cellular processes, such as nuclear transport, transcriptional regulation, apoptosis, and protein stability.	Protein modulation
THBS4	*Thrombospondin 4.* The protein belongs to the thrombospondin protein family; a family of adhesive glycoproteins that mediate cell-to-cell and cell-to-matrix interactions.	Adhesion<br>Cell-matrix, cell-cell
TSSK4	*Testis specific serine kinase 4.* This testis-specific serine/threonine kinase family member is thought to stimulate the CREB/CRE responsive pathway through phosphorylation of the cAMP responsive element binding protein transcription factor.	CREB<br>Transcription
VNN1	*Vanin 1.* This member of the vanin protein family is likely a GPI-anchored cell surface molecule.	Cell surface molecule

Table S10. A comparison of the constitutive extracellular protein release by human MSCs and osteoblasts; a summary of the proteins with significantly increased release by MSCs. The table presents the GO terms (identity/name) the number (number of proteins included/number of different proteins) and percentage of included proteins (number of proteins in the term/number of proteins included in the analysis, the corresponding percentage), the *p*-value and the false discovery rate (FDR). The GO analysis was performed using aGOtool (Main classification: Extracellular matrix, adhesion and proteases).

GO term		Numbers (percent)	*p*- value	FDR
**Biological process**				
...GO:0043062	Extracellular structure organization	47/185 (25.4)	4.70E-10	1.81E-06
.GO:0032502	Developmental process	105/185 (56.8)	1.18E-07	0.000151
..GO:0007155	Cell adhesion	46/185 (24.9)	9.79E-07	0.000944
...GO:0030574	Collagen catabolic process	19/185 (10.3)	1.78E-06	0.00137
....GO:0051093	Negative regulation of developmental process	32/185 (17.3)	1.22E-05	0.00524
....GO:0010810	Regulation of cell-substrate adhesion	20/185 (10.8)	2.33E-05	0.00898
.....GO:0030334	Regulation of cell migration	37/185 (20.0)	3.22E-05	0.0104
.....GO:0051960	Regulation of nervous system development	26/185 (14.1)	6.32E-05	0.0174
....GO:0045595	Regulation of cell differentiation	49/185 (26.5)	8.05E-05	0.0187
**Cell compartment** _				
..GO:0031012	Extracellular matrix	66/185 (35.7)	5.36E-11	2.51E-08
..GO:0005615	Extracellular space	77/185 (41.6)	3.53E-09	8.26E-07
..GO:0044420	Extracellular matrix component	27/185 (15.0)	1.01E-07	1.58E-05
...GO:0005581	Collagen trimer	19/185 (10.3)	1.59E-07	1.86E-05
....GO:0005788	Endoplasmic reticulum lumen	37/185 (20.0)	9.74E-07	9.12E-05
.GO:0005576	Extracellular region	81/185 (43.8)	1.46E-06	0.000114
..GO:0009986	Cell surface	29/185 (15.7)	4.67E-05	0.00243
**Uniprot**				
.UPK:0732	Signal	103/188 (54.8)	1.14E-11	3.30E-09
.UPK:0964	Secreted	81/188 (43.1)	2.89E-10	4.17E-08
..UPK:0272	Extracellular matrix	41/188 (21.8)	4.63E-10	4.46E-08
.UPK:0325	Glycoprotein	101/188 (53.7)	6.92E-10	5.00E-08
.UPK:0176	Collagen	19/188 (10.1)	4.71E-08	2.72E-06
.UPK:1015	Disulfide bond	86/188 (45.7)	2.63E-07	1.27E-05
.UPK:0130	Cell adhesion	30/188 (16.0)	7.38E-06	0.000305
.UPK:0379	Hydroxylation	20/188 (10.6)	2.63E-05	0.00095
UPK:9994	Domain	138/188 (73.4)	7.12E-05	0.00229
.UPK:0677	Repeat	85/188 (45.2)	0.000164	0.00474
.UPK:0106	Calcium	35/188 (18.6)	0.00026	0.00683
.UPK:0621	Polymorphism	136(188 (72.3)	0.000295	0.0071
.UPK:0358	Heparin-binding	12/188 (6.4)	0.00157	0.035
**KEGG**				
KEGG:04974	Protein digestion and absorption	13/93 (14.0)	1.69E-05	0.00176
KEGG:04512	ECM-receptor interaction	17/93 (18.3)	2.25E-05	0.00176
KEGG:04510	Focal adhesion	20/93 (21.5)	0.000124	0.00431
KEGG:05146	Amoebiasis	13/93 (14.0)	0.000855	0.0222

Table S11. MSC or osteoblast proteins included in the network Platelet degranulation (Figure 3). Green color indicates proteins that are increased in osteoblasts. The summary is based on the Gene database.

IDENTITY	DESCRIPTION	KEY WORDS
ALDOA	*Aldolase, fructose-bisphosphate A.* This member of the class I fructose-bisphosphate aldolase protein family is glycolytic enzyme that catalyzes the reversible conversion of fructose-1,6-bisphosphate to glyceraldehyde 3-phosphate and dihydroxyacetone phosphate. Mutations in this gene have been associated with Glycogen Storage Disease XII, an autosomal recessive disorder associated with hemolytic anemia. Disruption of this gene also plays a role in the progression of multiple types of cancers. The gene shows high expression in several tissues including bone marrow, spleen and lymph nodes.	Metabolism<br>Hemolytic anemia<br>Cancer
APOC3	*Apolipoprotein C3.* This gene encodes a protein component of triglyceride (TG)-rich lipoproteins (TRLs) including very low density lipoproteins (VLDL), high density lipoproteins (HDL) and chylomicrons. The encoded protein plays a role in role in triglyceride metabolism.	Metabolism
APP	*Amyloid beta precursor protein.* This cell surface receptor and transmembrane precursor protein is cleaved by secretases to form a number of peptides. Some of these peptides are secreted and can bind to the acetyltransferase complex APBB1/TIP60 to promote transcriptional activation, while others form the protein basis of amyloid plaques. It is expressed in a wide range of organs/tissues, including bone marrow, lympho nodes and spleen.	Cell surface<br>Transmembrane protein<br>Transcription
CLU	*Clusterin.* The protein is a secreted chaperone that also can be detected in the cell cytosol. It has been suggested to be involved in several basic biological events such as cell death and tumor progression. It is expressed in several organs/tissues with low expression in normal bone marrow/spleen but shows higher expression in the lymph nodes.	Chaptrone<br>Extracellular<br>Cancer
ENO1	*Enolase 1.* This gene encodes alpha-enolase that functions as a glycolytic enzyme. Alternative splicing of this gene results in a shorter isoform that has been shown to bind to the c-myc promoter and function as a tumor suppressor. The gene shows high expression in several tissues including bone marrow, spleen and lymph nodes.	Metabolism<br>Glycolytic enzyme<br>Tumor suppressor
FN1	*Fibronectin 1.* Fibronectin is a glycoprotein present in a soluble dimeric form in plasma, and in a dimeric or multimeric form at the cell surface and in extracellular matrix. The encoded preproprotein is proteolytically processed to generate the mature protein. Fibronectin is involved in cell adhesion and migration processes including embryogenesis, wound healing, blood coagulation, host defense, and metastasis. Low expression of this gene is seen in both normal bone marrow and lymphoid organs.	Extracellular matric<br>Cell migration
GAS6	*Growth arrest specific 6.* This gamma-carboxyglutamic acid (Gla)-containing protein is thought to be involved in the stimulation of cell proliferation; the gene is frequently overexpressed in many cancers and has been implicated as an adverse prognostic marker. The gene is expressed in many organs, but the expression in normal bone marrow is low.	Cell proliferation<br>Cancer
GPC1	*Glypicin 1.* Cell surface heparan sulfate proteoglycans are composed of a membrane-associated protein core substituted with a variable number of heparan sulfate chains. Members of the glypican-related integral membrane proteoglycan family contain a core protein anchored to the cytoplasmic membrane via a glycosyl phosphatidylinositol linkage. These proteins may play a role in the control of cell division and growth regulation. The expression in normal bone marrow is low.	Cell surface<br>Growth regulation
LRP1	*LDL receptor related protein 1.* This gene encodes a member of the low-density lipoprotein receptor family of proteins. The encoded preproprotein is proteolytically processed. This receptor is involved in several cellular processes, including intracellular signaling, lipid homeostasis, and clearance of apoptotic cells. The gene shows low expression in normal bone	Lipid metabolism

	marrow, spleen and lymph nodes.	
PLAU	*Plasminogen activator urokinase.* This gene encodes a secreted serine protease that converts plasminogen to plasmin. The encoded preproprotein is proteolytically processed to generate A and B polypeptide chains. The gene is expressed in many tissues/organs, including normal bone marrow.	Protease<br>Coagulation
PLTP	*Phospholipid transfer protein.* The encoded protein is one of at least two lipid transfer proteins found in human plasma. It transfers phospholipids from triglyceride-rich lipoproteins to high density lipoprotein (HDL). In addition to regulating the size of HDL particles, this protein may be involved in cholesterol metabolism. The gene is expressed I many organs/tissues.	Lipid metabolism
SERPINE1	Serpin family E member 1. This serine proteinase inhibitor is the principal inhibitor of tissue plasminogen activator (tPA) and urokinase (uPA). It has low expression in many organs/tissues, also normal bone marrow and lymph nodes/spleen.	Serine protease inhibitor
SERPING1	*Serpin family G member 1.* This highly glycosylated protein inhibits activated C1r and C1s of the first complement component and thus regulates complement activation.	Complement inhibition
SRGN	*Serglycin.* This protein is a hematopoietic cell granule proteoglycan. Proteoglycans stored in the secretory granules of many hematopoietic cells also contain a protease-resistant peptide core, which may be important for neutralizing hydrolytic enzymes. This encoded protein is associated with the macromolecular complex of granzymes and perforin. The gene is highly expressed in normal bone bone marrow, higher than in many other organs/tissues.	Secretory granules
TGFB1	*Transforming growth factor beta 1.* This cytokine binds various TGF-beta receptors leading to recruitment and activation of SMAD family transcription factors that regulate gene expression. The encoded preproprotein is proteolytically processed. The mature peptide may form heterodimers with other TGFB family members. This encoded protein regulates cell proliferation, differentiation and growth, and can modulate expression and activation of other growth factors including interferon gamma and tumor necrosis factor alpha. It is expressed in several tissues/organs.	Cytokine
THBS1	*Thrombospondin 1.* The protein is a subunit of a disulfide-linked homotrimeric protein. It is an adhesive glycoprotein that mediates cell-to-cell and cell-to-matrix interactions. This protein can bind to fibrinogen, fibronectin, laminin, type V collagen and integrins alpha-V/beta-1, and it has roles in platelet aggregation, angiogenesis, and tumorigenesis. The gene is expressed in many organs/tissues, including normal bone marrow, spleen and lymph nodes.	Platelet aggregation<br>Adhesion<br>Angiogenesis<br>Carcinogenesis

Table S12. Proteins showing significantly different release by osteoblasts derived from male and female donors. Positive FC values (red color) indicate higher levels in female donors, negative FC (blue color) indicates higher levels in male donors. The table presents the gene identity/name, fold change (FC) and the p-value for the Welch's T-test.

Gene	Fold change	p-value
COL12A1	3,9172	0,00583214
ADM	3,56355	0,0113848
ALCAM	3,50705	0,0439449
AEBP1	3,21815	0,00991128
NPC2	3,05285	0,00237781
CLU	3,0483	0,0107678
ECM1	2,9865	0,00273242
GREM1	2,8583	0,0111503
IGFBP3	2,8274	0,0146121
ATP6AP1	2,77635	0,0471022
SPOCK1	2,65585	0,0198271
CTSB	2,64075	0,00447139
FAM3C	2,62215	0,0333052
CLEC3B	2,5564	0,0352516
EFEMP2	2,5213	0,0207342
PSAP	2,4455	0,0195447
CSTB	2,4262	0,0357297
CTSA	2,41495	0,0218968
CTGF	2,4117	0,0231387
COL3A1	2,3762	0,0153434
BLMH	2,30145	0,0317783
GOLM1	2,2475	0,0197072
CD248	2,162	0,00997178
CD109	2,12685	0,0487072
THBS2	2,026	0,0317175
MARCKS	1,97045	0,039555
LOXL2	1,96725	0,0327348
CST3	1,79495	0,0428307
CLEC11A	1,78445	0,00510302
C1R	1,7734	0,013496
TIMP2	1,73575	0,013516
PTGDS	1,72685	0,0145427
CTSL	1,68405	0,0381368
TIMP1	1,61435	0,0238989
CYR61	1,55095	0,00171851
PCOLCE	1,5505	0,0236346
FSTL1	1,3911	0,0304739
LGALS3BP	1,3438	0,0226022
NUCB1	0,9058	0,0446702
HNRNPA3	-1,3532	0,0265133

CMPK1	-1,447	0,0363437
C3	-1,6425	0,0226989
F10	-1,96495	0,0332557
AIFM1	-2,14105	0,0361654
GALNT10	-2,20535	0,0309268
DDAH2	-2,24845	0,0192639
HBA1	-2,90705	0,0155052
HIST1H1E	-2,9644	0,011088

Table S13. Expression of proteins included in the GO term exosomes by human MSCs and osteoblasts. The proteins are listed in alphabetic order based on their gene identity. Proteins only expressed by MSCs are colored in blue whereas proteins expressed only by osteoblasts are colored in green. Proteins belonging to the 100 top-ranked exosomal proteins (http:/exocarta.org/exosome_markers_new) are written in red, the top-25 ranked are in addition marked in **bold**.

ABHD14B	APOA1BP	BPI	CDC42	CSTB	EIF3I	GALNS
ACAT2	APOB	BSG	CDH11	CTSA	EIF4A1	GANAB
ACLY	APOC3	BTD	CDH13	CTSB	EIF6	**GAPDH**
**ACTB**	APOE	C1R	CFB	CTSC	ELANE	GARS
ACTBL2	APP	C2	CFD	CTSD	ELAVL1	GAS6
ACTC1	APRT	C3	CFH	CTSF	EMILIN1	GBA
ACTG1	ARF1	C4A	CFI	CTSG	**ENO1**	GBE1
ACTN1	ARF4	C4B	CFL1	CTSL	ENO2	GDI2
ACTN4	ARHGAP1	CA1	CFL2	CTSZ	ERAP1	GGCT
ACTR1A	ARHGDIA	CA2	CHI3L1	CUTA	ERP44	GGH
ACTR2	ARHGDIB	CAB39	CHID1	DAG1	ESD	GLO1
ACTR3	ARL3	CACNA2D1	CLEC3B	DBI	EXT2	GLOD4
ADAM10	ARPC1A	CACYBP	CLIC1	DBNL	EZR	GLRX
ADAM9	ARPC1B	CAD	CLIC4	DCD	FABP1	GM2A
ADH5	ARPC2	CALR	**CLTC**	DCPS	FABP3	GNAI2
AEBP1	ARPC3	CAND1	CLU	DCTN2	FABP5	GNB1
AGRN	ARPC4	CANT1	CMPK1	DDAH1	FAH	GNB2
AGT	ARPC5	CANX	CNDP2	DDAH2	FAM129B	GNB2L1
AHCY	ARSA	CAP1	CNTN1	DDB1	FAM20C	GNPDA1
AHNAK	ASAH1	CAPG	COL1A2	DDT	FAM3C	GNPTG
AHSA1	ATIC	CAPN1	COL4A2	DEFA3;DEFA1	FAM49B	GNS
AK2	ATP5A1	CAPN2	COL6A1	DNASE2	**FASN**	GOT1
AKR1A1	ATP5B	CAPNS1	COL6A2	DNPH1	FAT1	GOT2
AKR1B1	ATP6AP1	CAPZA1	COL6A2	DPP3	FBLN1	GP1BA
AKR1C1	ATP6AP2	CAPZA2	COL6A3	DPP7	FBLN1	GPC1
ALAD	ATP6V1A	CAPZB	COL12A1	DPYSL2	FBLN5	GPI
ALCAM	ATP6V1B2	CAT	COL18A1	DSTN	FBP1	GRB2
**ALDOA**	ATP6V1E1	CBR1	COMP	DYNC1H1	FERMT3	GRN
ALDOC	ATP6V1F	CCT2	CORO1A	ECH1	FH	GSN
ALPL	ATP6V1G1	CCT3	CORO1B	ECM1	FKBP4	GSR
ALYREF	ATRN	CCT4	COTL1	EDIL3	FKBP5	GSS

ANGPT1	AXL	CCT5	CPE	**EEF1A1**	FLNA	GSTO1
ANGPTL2	AZU1	CCT6A	CPQ	EEF1G	FLNB	GSTP1
ANP32B	B2M	CCT7	CREG1	**EEF2**	FLRT2	GAA
ANPEP	B4GALT1	CCT8	CRK	EFEMP1	FN1	H2AFV
ANXA1	B4GAT1	CD14	CRYZ	EFEMP2	FN1	H2AFY
**ANXA2**	BASP1	CD248	CSE1L	EHD1	FN1	H3F3A
**ANXA5**	BAX	CD44	CSPG4	EHD2	FSCN1	HBA1
ANXA6	BGN	CD59	CSRP1	EIF2S1	FSTL1	HBB
APEH	BLMH	**CD81**	CST3	EIF2S3	FUCA1	HDGF
APLP2	BLVRB	CDC37	CST6	EIF3H	FUCA2	HDHD2
HEBP1	IGFBP7	MTAP	PCNA	PROCR	PROCR	RHOA
HEBP2	ILF3	MVP	PCOLCE	PROS1	PROS1	RHOG
HEXA	IMPDH2	MXRA5	**PDCD6IP**	PRSS23	PRSS23	RNASET2
HEXB	IQGAP1	MXRA8	PDIA3	PRTN3	PRTN3	RNH1
HINT1	IQGAP2	MYH14	PDIA6	PSAP	PSAP	RNPEP
HIST1H2AC	ISLR	MYH9	PDXK	PSAT1	PSAT1	RP2
HIST1H2AJ	ITGAM	MYL12A	PEBP1	PSMA1	PSMA1	RPE
HIST1H2BM	ITGB1	MYL6	PEPD	PSMA2	PSMA2	RPL10A
HIST1H3A	ITGB2	MYO1G	PFAS	PSMA3	PSMA3	RPL12
HIST1H4A	ITM2B	MYOF	PFKP	PSMA4	PSMA4	RPL23
HIST2H2AC	KPNB1	NACA	PFN1	PSMA5	PSMA5	RPL23A
HIST2H2BE	KRT6B	NAGK	PFN2	PSMA6	PSMA6	RPL3
HIST2H3A	LAMA4	NAGLU	PGAM1	PSMA7	PSMA7	RPL30
HLA-DRA	LAMB1	NAMPT	PGD	PSMB1	PSMB1	RPL4
HMCN1	LAMB2	NANS	**PGK1**	PSMB2	PSMB2	RPL5
HNMT	LAMC1	NAPA	PGLS	PSMB3	PSMB3	RPLP0
HNRNPA1	LAMP1	NAPRT	PGM1	PSMB4	PSMB4	RPLP2
HNRNPA2B1	LAMP2	NARS	PGM2	PSMB5	PSMB5	RPSA
HNRNPC	LAMTOR3	NCL	PHB	PSMB6	PSMB6	RPS2
HNRNPD	LCP1	NEDD8	PHPT1	PSMB8	PSMB8	RPS3
HNRNPK	**LDHA**	NID1	**PKM**	PSMB9	PSMB9	RPS3A
HNRNPL	LDHB	NID2	PLAU	PSMD2	PSMD2	RPS4X
HNRNPM	LEPRE1	NIT2	PLBD2	PSME1	PSME1	RPS5
HNRNPR	LGALS1	NME1	PLEC	PSME2	PSME2	RPS8
HP	LGALS3	NME2	PLOD1	PTBP1	PTBP1	RPS11
HPRT1	LGALS3BP	NPC2	PLOD2	PTGDS	PTGDS	RPS13
HPX	LGMN	NPEPPS	PLOD3	PTPN6	PTPN6	RPS14
**HSP90AB1**	LMAN2	NQO2	PLXNA1	PTPRC	PTPRC	RPS15A
HSP90AB2P	LRRC15	NT5E	PLXNB2	PTPRG	PTPRG	RPS18
HSP90B1	LSP1	NUCB1	PLAA	PXDN	PXDN	RPS19
**HSP90AA1**	LTA4H	NUCB2	PNP	PYGB	PYGB	RPS20
HSPA1B	LTBP2	NUDT5	PPA1	PYGL	PYGL	RPS26
HSPA4	LTBP3	NUMA1	PPIA	QPCT	QPCT	RPS27A
HSPA5	LTF	NUTF2	PPIB	QSOX1	QSOX1	RPS28
HSPA6	LUM	OGN	PPIC	RAB10	RAB1A	RSU1
**HSPA8**	LYPLA2	OMD	PPP1CA	RAB11A	RAB1B	RUVBL1
HSPA9	LYZ	OTUB1	PPP1R7	RAB11B	RAB2A	RUVBL2
HSPB1	MAN1A1	P4HB	PPP2CA	RAB14	RAB5C	S100A4

HSPD1	MAN2B1	PA2G4	PPP2R1A	RAB1A	RAB6A	S100A6
HSPE1	MARCKS	PABPC1	PPT1	RAB1B	RAB7A	S100A9
HSPG2	MDH1	PABPN1	PRCP	RAB2A	RAB8B	S100A10
HSPH1	MDH2	PAFAH1B1	PRDX1	RAB5C	RAB10	S100A11
HTRA1	MFGE8	PAFAH1B2	PRDX2	RAB6A	RAB11A	S100P
IDH1	MGP	PAICS	PRDX4	RAB7A	RAB11B	SARS
IDH2	MIF	PAM	PRDX5	RAB8B	RAB14	SBSN
IGF2R	MMP9	PARK7	PRDX6	RAN	RAN	SCN11A
IGFALS	MPO	PCBP1	PRELP	RBMX	RBMX	SCPEP1
IGFBP2	MSN	PCMT1	PRNP	RDX	RDX	SDC1
SDC4	TF	VTA1				
**SDCBP**	TGFBI	WARS				
SDF4	TGM2	WDR1				
SEC13	THBS1	WNT5B				
SELENBP1	THBS4	YBX1				
SEMA3C	THRAP3	YWHAB				
SEPP1	THY1	**YWHAE**				
SEPT2	TIMP1	YWHAG				
SEPT7	TKT	YWHAH				
SERBP1	TLN1	YWHAQ				
SERPINB1	TPI1	**YWHAZ**				
SERPINB6	TPM3					
SERPINB8	TPM3					
SERPINE1	TPM4					
SERPINF1	TPM4					
SERPING1	TPP1					
SH3BGRL	TPT1					
SH3BGRL3	TSTA3					
SIRPA	TTN					
SLC1A5	TUBB					
SLC2A1	TUBB3					
SLC3A2	TUBB4B					
SLC4A1	TUBB8					
SLC9A3R1	TUFM					
SMS	TWF2					
SND1	TXN					
SNRPD2	TXNDC5					
SOD1	TXNDC17					
SOD3	TXNRD1					
SORD	TXNRD1					
SPON2	UBA1					
SPP1	UBE2D2					
SPP1	UBE2I					
SPTAN1	UBE2N					
SPTBN1	UBE2V1					
SRPR	UGGT1					
SRSF7	UGP2					
ST13	USP14					

STAU1	VAMP8
STMN1	VASN
STX7	VASP
SUB1	VAT1
SUMO2	VCAM1
SYNCRIP	VCL
TAGLN2	VCP
TALDO1	VIM
TARS	VPS35
TCP1	VPS4B

Table S14. Expression of proteins included in the GO term proteases by human MSCs and osteoblasts. The proteins are listed in alphabetic order based on their gene identity. Proteins only expressed by MSCs are colored in blue whereas proteins expressed only by osteoblasts are colored in green.

ADAM10	COL7A1	HBB	PEBP1	SERPINB2
ADAM19	COMP	HGF	PEPD	SERPINB6
ADAM9	COPS4	HMGB2	PLAU	SERPINB7
ADAMTS1	CPA4	HP	PRCP	SERPINB8
ADAMTS2	CPE	HTRA1	PRDX4	SERPINB10
ADAMTS4	CPQ	INS-IGF2	PRNP	SERPINE1
ANPEP	CST3	ITGAM	PROS1	SERPINE2
ANXA2	CST6	ITGB1	PRSS23	SERPINF1
APEX1	CSTB	ITM2B	PRTN3	SERPING1
APLP2	CTSA	LDLR	PSAP	SET
APP	CTSB	LFNG	PSMA1	SET
ARF1;ARF3	CTSC	LGMN	PSMA2	SRGN
ARSA	CTSD	LRP1	PSMA3	STAT1
ATP5A1	CTSF	LTA4H	PSMA4	SUMO1
ATP5B	CTSG	LTF	PSMA5	SUMO2
ATP6AP2	CTSK	MBTPS1	PSMA6	TFPI
ATPIF1	CTSL	METAP2	PSMA7	TGFB1
AZU1	CTSS	MIF	PSMB1	TIMP1
BIN1	CTSZ	MMP1	PSMB2	TIMP2
BLMH	DCD	MMP2	PSMB3	TPP1
BMP1	DDI2	MMP11	PSMB4	TPP2
C1QBP	DLD	MMP14	PSMB5	TRIM28
C1R	DLST	MMP8	PSMB6	TTN
C1S	DPP3	MMP9	PSMB7	TXN2
C2	DPP7	MRC2	PSMB8	UBXN1
CAPN1	ECM1	MYDGF	PSMB9	UCHL1
CAPN2	EIF4G1	NARS	PSMB10	UCHL3
CAPNS1	ELANE	NEDD8	PSMC4	USP14
CASP3	ERAP1	NPEPPS	PSMD2	USP15
CAST	F10	NPM1	PSMD4	USP48
CD109	FAP	OS9	PSME1	USP5
CFB	FLNC	OTUB1	PSME2	VCP
CFD	FN1	P4HB	PSME3	VPS26A

CFH	FN1	PAMR1	PYCARD	YBX1
CFI	FN1	PAPPA	RAD23A	
CLTC	G3BP1	PARK7	RHOA;RHOC	
CNDP2	G3BP2	PCOLCE	RNPEP	
COL1A1	GAPDH	PCSK5	SART3	
COL1A2	GRN	PDGFRB	SCPEP1	
COL3A1	HABP2	PDIA3	SERPINA9	
COL6A3	HBA1	PDIA6	SERPINB1	

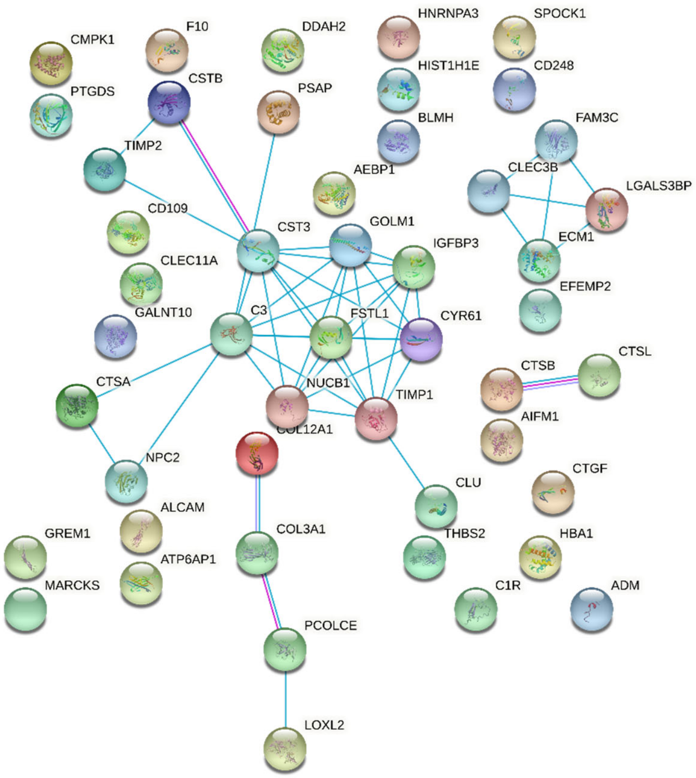

Figure S1. Network analysis of proteins released by osteoblasts derived from healthy donors and showing significant differences when comparing male and female donors. See Table s14 for additional information (p-values, fold change, increased/decreased in female donors).
